# Supplementary material for: Unraveling the molecular basis of substrate specificity and halogen activation in vanadium-dependent haloperoxidases
Source: Nat Commun. 2025 Feb 28;16:2083. doi: 10.1038/s41467-025-57023-1 (PMC11871015; doi:10.1038/s41467-025-57023-1)
Supplement: Supplementary file 1 — Supplementary Information [file 41467_2025_57023_MOESM1_ESM.pdf]

Supplementary Information

Unraveling the Molecular Basis of Substrate Specificity and Halogen Activation in Vanadium-Dependent Haloperoxidases

P. Zeides,[a,b,+] K. Bellmann-Sickert,[b,+] Ru Zhang,[b] C. J. Seel,[a] V. Most,[c] C. T. Schoeder,[c] M. Groll,[d] T. Gulder [a,b,e,f*]

[a]Biomimetic Catalysis, Catalysis Research Center, TUM School of Natural Sciences, Technical University of Munich, Lichtenbergstrasse 4, 85748 Garching, Germany

[b]Institute of Organic Chemistry, Faculty of Chemistry and Mineralogy, Leipzig University, Johannisallee 29, 04103 Leipzig, Germany

[c]Institute for Drug Discovery, Leipzig University, Faculty of Medicine, Liebigstr. 19, 04103 Leipzig

[d] Department of Bioscience, Center for Protein Assemblies, TUM School of Natural Sciences, Technical University of Munich, Ernst-Otto-Fischer Strasse 8, 85748 Garching, Germany

[e]Organic Chemistry, Saarland University, 66123 Saarbruecken, Germany

[f]Synthesis of Natural-Product Derived Drugs, Helmholtz Institute for Pharmaceutical Research Saarland (HIPS) Helmholtz Centre for Infection Research (HZI), 66123 Saarbrücken, Germany

e-mail: tanja.gulder@uni-leipzig.de

[+]authors have contributed equally

TABLE OF CONTENTS

1. General Information 4
2. Enzyme Production 5
2.1 Cloning and site-directed mutagenesis 5
2.2 Activity screening of *Am*VHPO single-point mutants 6
2.3 Expression and purification 8
3. Enzyme Activity of single-point mutants 9
3.1 Monochlorodimedone assay 9
3.2 Phenol red assay 10
3.3 1,3,5-Trimethoxybenzene (7, TMB) conversion by LC analysis 13
4. Enzyme Activity of Double Mutants 16
4.1 Monochlorodimedone assay 16
4.2 Phenol red assay 17
5. Enzyme kinetics 19
6. Competition Assay for Chlorination of MCD (3) and TMB (7) 22
7. Computational screening for mutation sites close to the vanadate binding site 25
8.1 Structure preparation 25
7.2 Sequence design 25
7.3 ΔΔG calculations 25
7.4 Visualization 25
7.5 Molecular dynamics (MD) simulations 28
8. Crystallization 30
8.1 Crystallization of *Am*VHPO variants 30
8.2 Structure determination of *Am*VHPO variants 30
8.3 Electron density maps 32
8.4 Comparison of the possible loop regions in the *Am*VHPO variants 32
9. Comparison of the Structure and the Chlorination Activity of different VHPOs 33
10. Bromination and chlorination activity of corallina vhpos and their r→s mutants 37
10.1 Monochlorodimedone assay 37
10.2 TMB (7) conversion 38
11. Aromatic Chlorination Catalyzed by R425S-*Am*VHPO 39
11.1 General Procedure 39
11.2 Chlorination of 1,3,5-trimethoxybenzene (7) 40
11.3 Chlorination of thymol (S1) 42
11.4 Chlorination of 1,3-dimethoxybenzene (S3) 44
11.5 Chlorination of *N*,*N*-Dimethylaniline (S3) 46
11.6 Chlorination of 3,5-dimethyl-*1H*-pyrrole-2-carboxylic acid ethyl ester (S5) 48
11.7 Chlorination of 2-methylindole (S6) 50

11.8 Chlorination of 2,6-dimethoxypyridine (S7) 52
11.9 Chlorination of carbazole (S8) 54
11.10 Chlorination of 3,5,4‘-Trimethoxystilbene (S10) 55
12. Chemical chlorination 56
12.1 General Method 56
13. Sequence Alignment of VHPOs 58
14. References 60

1. GENERAL INFORMATION

Solvents used in reactions were p.A. grade. Solvents for chromatography were technical grade and distilled before use or purchased in purity grades appropriate for the selected method. Reagents were purchased at the highest commercial quality and used without further purification. Solvent mixtures are reported as volume/volume (*v*/*v*). NMR spectra were recorded on a Bruker AV500 spectrometer. The spectra were calibrated using residual undeuterated solvent as an internal reference ($CHCl_3$ @ 7.26 ppm, ^{1}H NMR, $CHCl_3$ @ 77.00 ppm). The following abbreviations (or combinations thereof) are used to explain the multiplicities: s = singlet, d = doublet, dd = doublet of doublets, t = triplet, dt = doublet of triplets, q = quartet, m = multiplet, br = broad. In addition, the following abbreviations are used: EtOAc = ethyl acetate, MeCN = acetonitrile, rt = room temperature, sat = saturated, MES = 2-(*N*-morpholino)ethanesulfonic acid, NaOAc = sodium acetate, Tris = Tris(hydroxymethyl)aminomethane, HEPES = 4-(2-hydroxyethyl)-1-piperazine-ethanesulfonic acid, TFA = trifluoroacetic acid, MCD = monochlorodimedone, TB = thymol blue, TMB = 1,3,5 trimethoxybenzene, PR = phenol red, NaOAc = sodium acetate, IPTG = isopropyl β-D-1-thiogalactopyranoside; Ni-NTA = nickel-nitrilotriacetic acid, LB = lysogeny broth. Mass spectra were determined on a Thermo LCQ fleet coupled with a Dionex UltiMate 3000 HPLC. HR-ESI-MS, HR-LC-ESI-MS, HR-APCI-MS, and HR-LC-APCI-MS mass spectra were recorded with a Thermo LTQ FT Ultra coupled with a Dionex UltiMate 3000 HPLC system. Gas chromatography (GC) was performed on a Varian 3900 gas chromatograph equipped with an HP-5 capillary column [length = 29.5 m] and a Varian Saturn 2000 as a mass spectrometer. Hydrogen was used as the carrier gas, and the constant-flow mode (flow rate = 1.8 mL min^{-1}) with a split ratio 1:20 was used. The following temperature-program was used: 60°C for 3 min, 15°C min^{-1} to 250°C, and 250°C for 5 min. *n*-Dodecanol served as an internal standard. High-performance liquid chromatography (HPLC) analysis was performed on a HITACHI Chromaster employing the following conditions: 150 x 4 mm Eurospher II (KNAUER®), 100 Å, 5 µm, C18, 25°C, flow rate: 1.0 mL min^{-1}, water (+0.01% TFA)/MeCN (+0.01% TFA) gradient: 90/10 → 0/100 in 13 min. Liquid chromatography-mass spectrometry (LC-MS) analysis was performed on an Agilent 1260 Infinity II system using an Agilent Poroshell 120 EC-C18 column (2.1 x 50 mm, particle size 2.7 µm). MeCN (solvent B) and 0.1% aqueous formic acid (solvent A) were solvents. The flow rate was 0.5 mL min^{-1}. The gradient was 2 to 70% B in A within 7 min. The detected mass range was 50 – 400 m/z. Phenol served as an internal standard. For protein purification, an ÄKTA FPLC system (GE Healthcare) with Histrap FF column (GE Healthcare) and Superdex S200 16/60 column (GE Healthcare) was used. Protein concentrations were determined using a Nanodrop 2000c spectrophotometer (Thermo Scientific) and the NanoPhotometer® N60 (Implen) spectrophotometer (ε(*Am*VHPO+His) =

65,780 $M^{-1}cm^{-1}$, ε(*Cp*VHPO+His) = 41495 $M^{-1}cm^{-1}$, ε(*Co*VHPO+His) = 41495 $M^{-1}cm^{-1}$). UV/Vis spectra were recorded on a BioTek® Eon spectrophotometer. The molecular weight of wild-type *Am*VHPO and mutants was 73.13 kDa, wild-type CpVHPO and mutants 66.37 kDa, and wild-type *Co*VHPO and mutants 66.51 kDa.

2. ENZYME PRODUCTION

2.1 Cloning and site-directed mutagenesis

The codon-optimized synthetic gene of the haloperoxidase I from *Acaryochloris marina* (wild-type *Am*VHPO), cloned into a pET28-based expression vector, was used as a template for site-directed mutagenesis experiments.[1] All mutants of *Am*VHPO were created by PCR using primers from STable 1. Genes for wildtype and mutants of *Co*VHPO and *Cp*VHPO were synthesized and cloned into pET28b(+) (Novagen, 69865-3) by Azenta and were confirmed *via* Sanger sequencing (GATC *Biotech*; Azenta).

STable 1 | Primer sequences used for site-directed mutagenesis of haloperoxidase I from *A. marina*

clone name	primer direction	sequence (5`−3`)
Wt-*Am*VHPO		
R425W	forward	GAACGTTGAACTTCTGAAACCAAACGGCTTTCAAACCGC
	reverse	TCGCGGTTTGAAAGCCGTTTGGTTTCAGAAGTTCAACGTTC
R425F	forward	GAACGTTGAACTTCTGAAAGAAAACGGCTTTCAAACCGCGAG
	reverse	CTCGCGGTTTGAAAGCCGTTTTCTTTCAGAAGTTCAACGTTC
R425S-*Am*VHPO		
R425S; E139G	forward	CGTGCCTGGGGTTCTGCGGGTGCAGGACT
	reverse	CAATTTCGCGCCATCGCCGCCTGCA
R425S; F401G	forward	CAGCAGGGCGGTGCGCATTTC
	reverse	TTTATCTTCAACGTCCGGCTTTTGA

2.2 Activity screening of *Am*VHPO single-point mutants

A site-saturated mutagenesis library of position R425 was ordered from ThermoFisher Scientific. The mixture of all mutants was heat-transformed into freshly prepared chemo-competent *E. coli* (BL21) cells and spread afterward on kanamycin-containing lysogeny broth (LB, Agarose Sigma, A9539, LB Medium Sigma, L3022) agar plates. A 2 mL well-plate with 94 single-picked colonies was incubated overnight (37 °C; 900 rpm). For expression of the enzyme, a second plate was prepared, inoculated, and incubated at 37 °C for 90 min before the expression (18 h, 18 °C, 900 rpm) was induced with IPTG (Sigma-Aldrich, I6758, 0.1 mM). After 18 h, the cells were centrifuged (2000 g; 10 min), resuspended in Tris (Fluka, T4661) pH 7 (50 mM Tris, 300 mM NaCl (Sigma, S9888), 10 mM imidazole (Carbolution, CC03002), 10% (v/v) glycerol (Alfa Aesar, A16205), and lyzed by the freeze-thaw method. The overnight cultures were also used to prepare a glycerol stock master plate. To detect all mutants with increased activity, the cultures were centrifuged (2000 g; 10 min) again, and an monochlorodimedone (3) (MCD, Carbolution, CC03081) assay was performed with the lysate. Therefore, 365 µL of a stock solution containing MCD (50 µM), MES (TCI, M0606) pH 6 (50 mM), KCl (Sigma, P4504) or KBr (Acros, AC196480010) (200mM), and Na_3VO_4 (Sigma, 450243) (300 µM) was mixed with 5 µL of the lysate. The measurement was started by adding 30 µL H_2O_2 (Acros, 202460010) (10mM) to the reaction mixture, and a decrease in absorbance was monitored at 290 nm and 30 °C for 30 min. Active mutants were identified by Sanger sequencing.

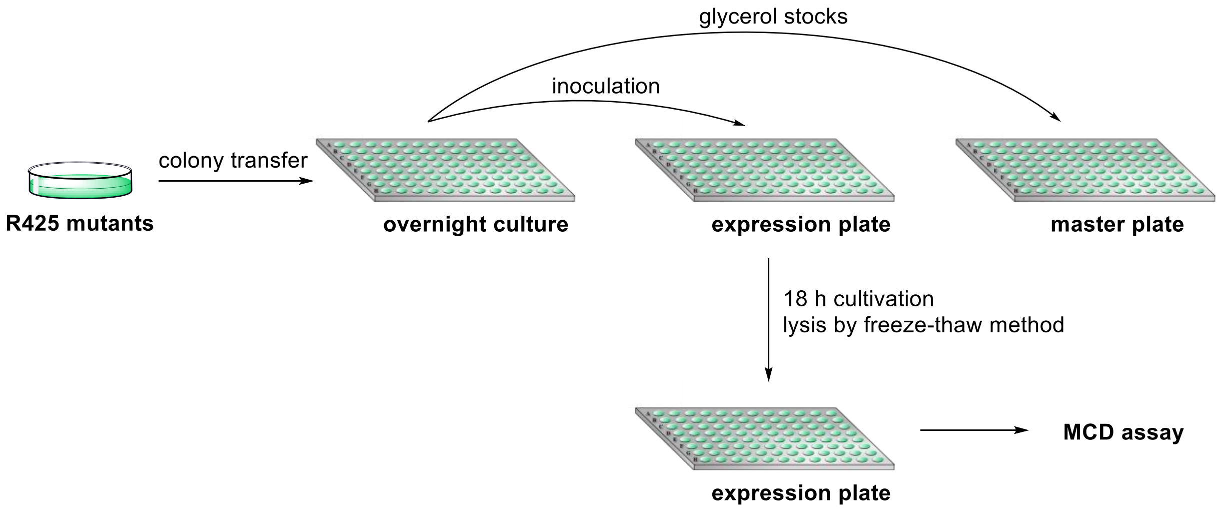

SFigure 1 | Expression and screening of site saturated mutagenesis library. Mutants were transformed into *E. coli* (BL21), expressed in 96 well plates and screened *via* monochlorodimedone assay.

STable 2 | Halogenation activities of relevant R425 mutants using the MCD assay.

R425 mutation	Conversion of MCD (3) after 126 s in the presence of Br^-	Conversion of MCD (3) after 618 s in the presence of Cl^-
wild-type	49.5 %, n = 1	7.53 %, n = 1
S	99.5 ± 0.2%, n = 4[b]	66.4 ± 10.7 %, n = 4[b]
K	98.4 %. n = 1	53.3 %, n = 1
P	17.8 ± 15.8 %, n = 2 [b]	43.8 ± 0.2 %, n = 2 [b]
I	43.3 %, n = 1	17.7 %, n = 1
A	27.0 ± 1.3 %, n = 2 [b]	29.2 ± 1.3 %, n = 2 [b]
Q	99.9 ± 0.1 %, n = 2 [b]	17.3 ± 5.0%, n = 2 [b]
G	90.6 %, n = 1	n. d., n = 1
T	100.6 %, n = 1	9.0 %, n = 1
E	99.4 ± 0.1 %, n = 2 [b]	41.5 ± 3.0 %, n = 2 [b]
C	99.2 ± 0.3 %, n = 2 [b]	18.2 ± 0.5 %, n = 2 [b]
D	83.5%, n = 1	2.81 ± 0.2 %, n = 1
W[c]	51.5 ± 15.1%, n = 3[d]	10.5 ± 0.7 %, n = 3[d]
F[c]	53.3 ± 9.6 %, n = 3[d]	n. d., n = 3[d]

[a] n. d. = not determined; [b] replicates are biological replicates; [c] W and F mutants were not found in the screening but tested separately as they are literature known mutants. Performance was examined by monochlorodimedone (MCD, 3) assay as described in 4.1. [d] replicates are technical replicates.

2.3 Expression and purification

Confirmed mutagenesis products were transformed into *E. coli* BL21 (DE3) using a standard heat shock protocol. Recombinant bacteria were grown on LB agar plates and amplified afterward in LB mediumat 37 °C to an optical density of 0.6 in the presence of kanamycin (50 µg/mL). The protein expression was induced by IPTG (1 mM), and the culture was incubated at 18 °C overnight. Cells were collected by centrifugation, washed with saline (0.9% NaCl), and resuspended in buffer A (50 mM Tris pH 7.0, 300 mM NaCl, 30 mM imidazole). Before sonication of the harvested cells, DNase I (ThermoFisher, EN0521) and protease inhibitors (PMSF, ThermoFisher, 36978) were added. After centrifugation, the supernatant was heated to 60 °C for 20 min to precipitate unwanted proteins. Soluble target proteins, carrying a His-Tag sequence, were purified by an Äkta purifier using a 5 mL Histrap FF column (GE Healthcare) and an imidazole gradient from 30 to 500 mM. After analyzing the protein peak fractions by SDS-PAGE, haloperoxidase fractions were concentrated by ultrafiltration and dialyzed against buffer B (20 mM Tris pH 7.0, 150 mM NaCl) for a size exclusion chromatography (S200 16/60 column, GE Healthcare). Target proteins were dialyzed against buffer C (50 mM Tris pH 7, 100 µM Na_3VO_4) for storage and further use.

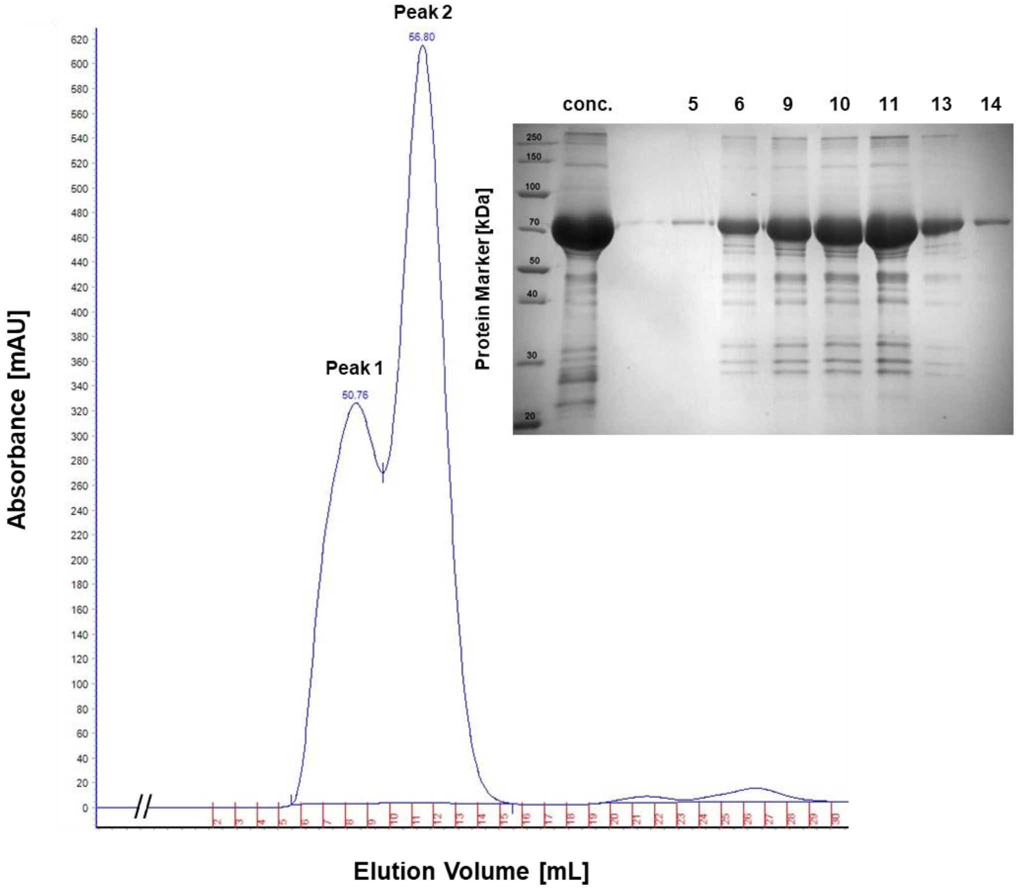

SFigure 2 | UV-absorbance chromatogram of R425S-*Am*VHPO. After size-exclusion chromatography using a Superdex S200 16/60 column, SDS PAGE analysis shows a clean enzyme in Peak 2 (fractions 10 – 14).

3. ENZYME ACTIVITY OF SINGLE-POINT MUTANTS

3.1 Monochlorodimedone assay

All reactions were performed in triplicates using 96-well microplates (Brand® F-bottom, UV-transparent, pureGrade™, 781600). A reaction mixture containing MES buffer pH 6 (50 mM), KCl (200 mM), Na_3VO_4 (1 µM), and the respective *Am*VHPO variant (4.4 µg/mL) was prepared, and MCD (50 µM; 1 mM stock solution in 2 M NaOAc (Fluka, 32319) was added. The volume per well was 270 µL. To start the measurement, 30 µL H_2O_2 (10 mM, final concentration 1 mM) was added last, and the decrease in absorption (290 nm) was monitored over time (30 min) at 30 °C by UV/Vis spectroscopy. Data was analyzed using Microsoft® Excel® 2019 MSO version 16.0.10366.20016 and OriginPro 2019 version 9.6.0.172.

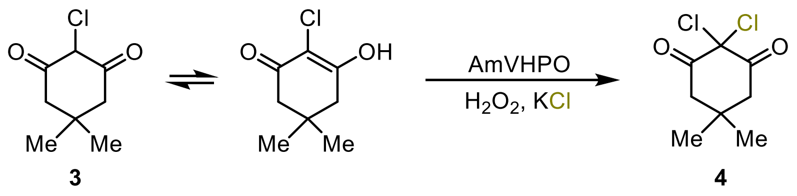

The fastest mutant, R425S, and one of the slowest chlorinating variants, R425D, together with the wild-type *Am*VHPO, are shown here for comparison.

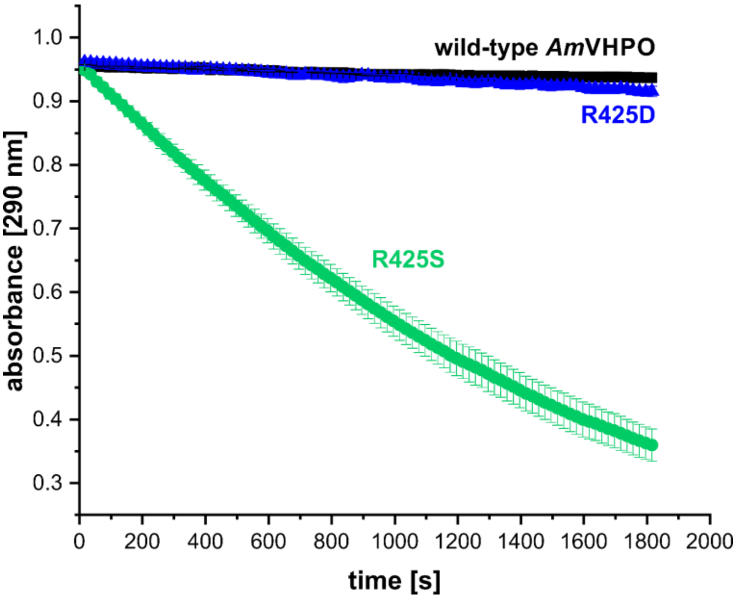

SFigure 3 | Comparison of VHPO variants via MCD assay for chlorination. Wild-type (black) and R425D variant (blue) showed almost no activity in the chlorination of monochlorodimedone, while for the R425S variant (green), a significant decrease in absorption is noticeable. Data are presented as mean ± SD for n = 3 independent experiments. Source data are provided as a Source Data file. MCD = monochlorodimedone.

Control experiments confirmed that the observed effect only occurred when all necessary components were present in the reaction mixture (H_2O_2, KCl, enzyme). When there is no halide present, a slight signal decrease is observed due to oxidation of MCD by H_2O_2. This is a known shunt pathway for haloperoxidases that can produce singlet oxygen in mildly acidic conditions.[1]

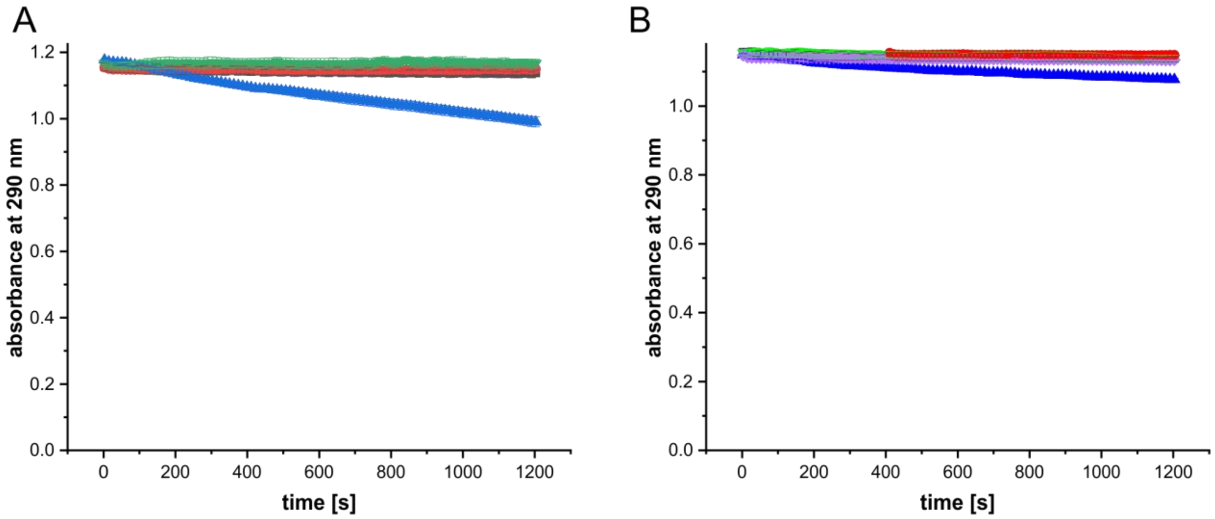

SFigure 4 | Control experiments MCD-assay. A: Wild-type controls; no enzyme in the presence of KCl (■), no enzyme in the presence of KBr (●), no halide (▲), no H_2O_2 in the presence of KBr (▼). B: R425S-*Am*VHPO controls; no enzyme in the presence of KCl (■), no enzyme in the presence of KBr (●), no halide (▲), no H_2O_2 in the presence of KBr (▼), no H_2O_2 in the presence of KCl (♦). Data are presented as mean ± SD for n = 3 independent experiments. Source data are provided as a Source Data file. *Am*VHPO = vanadium-dependent haloperoxidase from *Acaryochloris marina*.

3.2 Phenol red assay

All reactions were performed in triplicates using 96-well microplates (Brand® F-bottom, pureGrade™). Phenol red (Alfa Aesar, B21710) 14.3 µM; stock solution prepared in 100 mM NaOH) was added to a mixture of MES buffer pH 6 (50 mM), KX (142 mM, X = Cl, Br), Na_3VO_4 (300 µM) and the respective *Am*VHPO variant (for bromination 6.67 µg/mL; for chlorination 33.3 µg/mL). The reaction was started by the addition of H_2O_2 (10 mM), and the absorption (590 nm) was measured over time (bromination 10 min; chlorination 45 min) at 30 °C. Data were analyzed using Microsoft® Excel® 2019 MSO version 16.0.10366.20016 and OriginPro 2019 version 9.6.0.172.

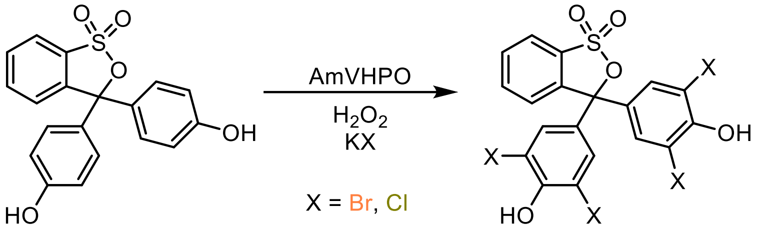

5
λ (pH 6) = 428 nm

6
λ = 590 nm

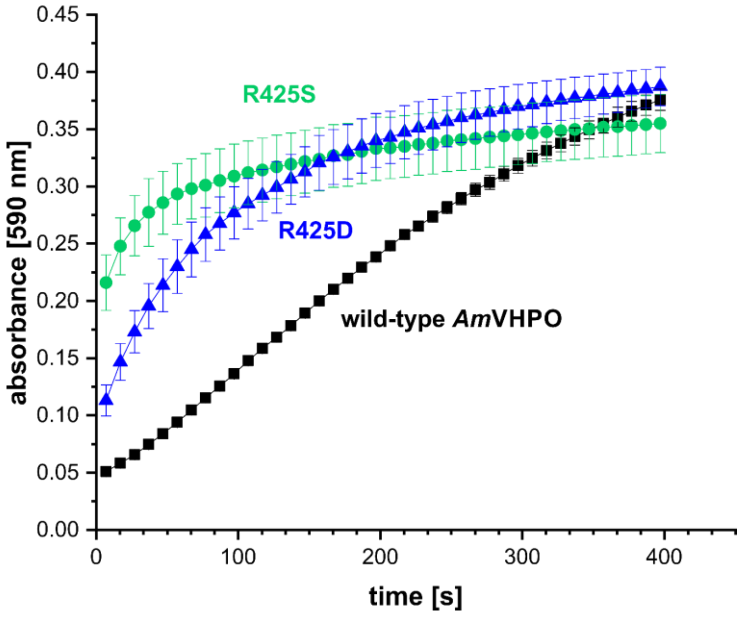

SFigure 5 | Activity assay for the bromination of phenol red. Comparison of bromination activity between the wild-type enzyme, the R425S, and the R425D variant. R425S showed a significantly higher reaction rate for the bromination of phenol red. Data are presented as mean ± SD for n = 3 independent experiments. Source data are provided as a Source Data file. *Am*VHPO = vanadium-dependent haloperoxidase from *Acaryochloris marina*.

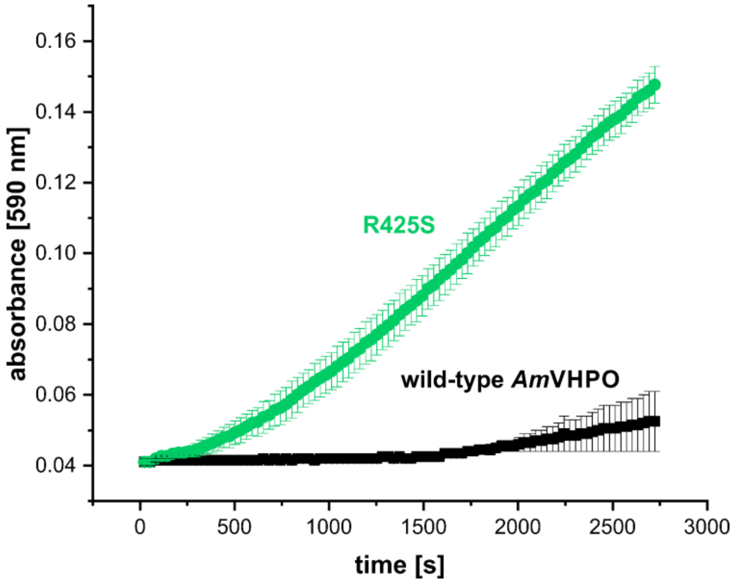

SFigure 6 | Activity assay for the chlorination of phenol red. Comparison of bromination activity between the wild-type enzyme, the R425S, and the R425D variant. R425S showed a significantly higher reaction rate for the bromination of phenol red. Data are presented as mean ± SD for n = 3 independent experiments. Source data are provided as a Source Data file. *Am*VHPO = vanadium-dependent haloperoxidase from *Acaryochloris marina*.

3.3 1,3,5-Trimethoxybenzene (7, TMB) conversion by LC analysis

High-performance liquid chromatography was performed on an Agilent 1260 Infinity II system. A reaction mixture containing MES buffer pH 6 (55 mM), KCl (550 mM), Na_3VO_4 (1.1 µM), 200 µM TMB (Sigma, 138827), and the respective *Am*VHPO variant (13.2 µg/mL) was prepared and incubated in a shaker at 30°C and 850 rpm. 110 µL H_2O_2 (10 mM, final concentration 1 mM) was added to start the reaction. 100 µL samples were taken at indicated time points. Reactions were quenched by adding 25 µL of sat. NaCl and 25 µL of sat. $(NH_4)_2SO_4$ (ThermoScientific, 10556792). 1.5 µL of a 3 mM phenol (Sigma, W3222318) stock solution (internal standard) was added, and samples were centrifuged and subjected to LC-MS analysis. Samples were analyzed using 2 to 70% or 80% acetonitrile (ThermoScientific, 10001334) gradient in 0.1% aqueous formic acid (Sigma, 5.330002) over 7 min. Chromatograms were extracted at 266 nm. Peak areas (AUC) were determined and referenced to the internal standard (phenol). t_R (phenol) = 2.7 min, t_R (TMB **7**) = 5.4 min, t_R (chlorinated TMB) = 5.7 min. Assays were performed as triplicates. Data were aquired using OpenLab CDS Acquisition version 2.6 and OpenLab CDS Analysis version 2.6. Data were analyzed using Microsoft® Excel® 2019 MSO version 16.0.10366.20016 and OriginPro 2019 version 9.6.0.172.

STable 3 | TMB conversion over time measured by LC analysis.

**Time [h]**	**TMB (7) conversion [%]**	
	**wt-*Am*VHPO**	**R425S-*Am*VHPO**
0	0	0
0.25	8.2 ± 0.9[a]	67.1 ± 2.9
0.5	4.3	71.3 ± 1.4
1	7.7 ± 2.0	71.4 ± 2.7
2	11.4 ± 0.5	76.4 ± 1.0
3	18.9 ± 2.3	83.7 ± 0.5
4	17.8 ± 2.1	85.6 ± 1.4
6	26.5 ± 1.6	91.8 ± 0.9
8	28.4 ± 1.7	93.7 ± 0.7

[a] Data are presented as mean ± SD for n = 3 independent experiments. TMB = 1,3,5-trimethoxybenzene.

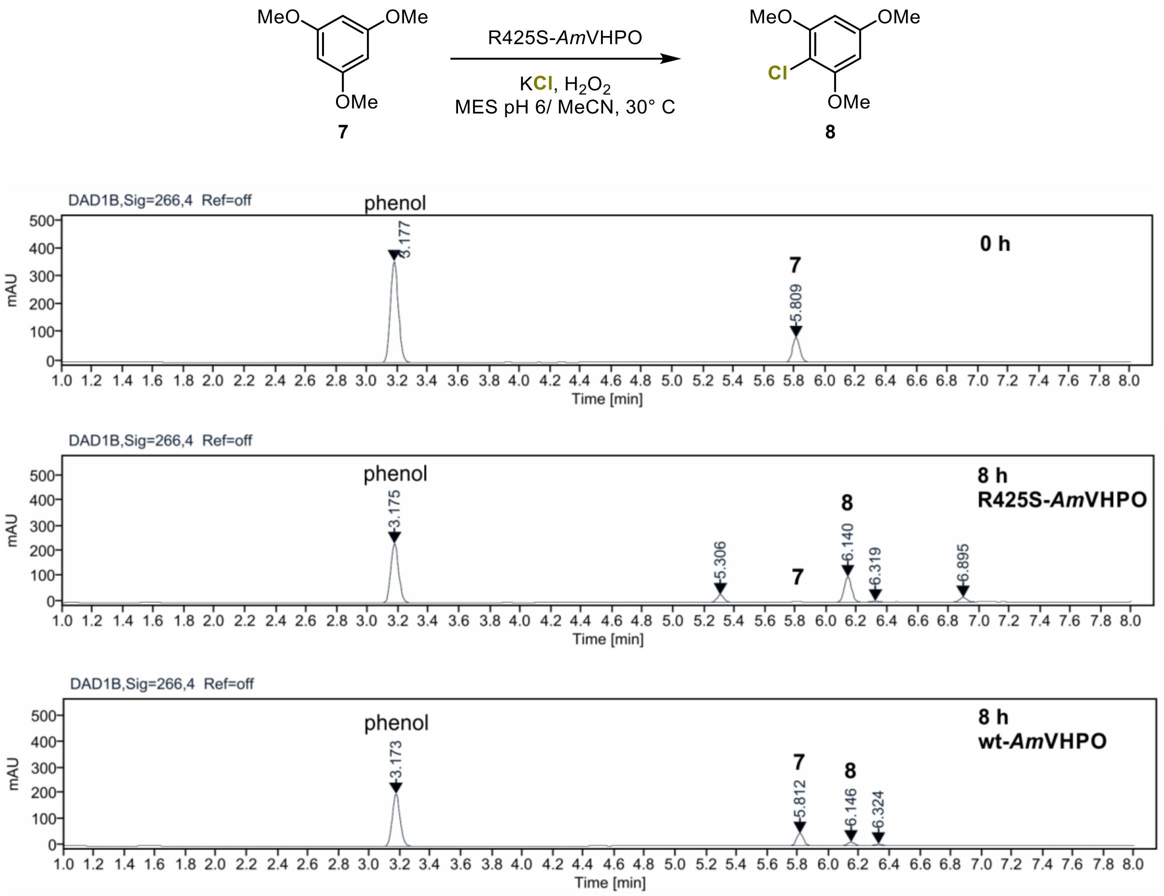

SFigure 7 | LC-Measurement of the wild-type- and R425S-*Am*VHPO catalyzed conversion of TMB (**7**) after 8h. *Am*VHPO = vanadium-dependent haloperoxidase from *Acaryochloris marina*.

Peaks at t_R = 5.31, 6.32, and 6.90 min do not show any isotope pattern typical of chlorination. While conversion using R425S-*Am*VHPO yielded ca 75% **8** after 8h, production of less than 10% **8** were detected by LC-MS using wild-type *Am*VHPO.

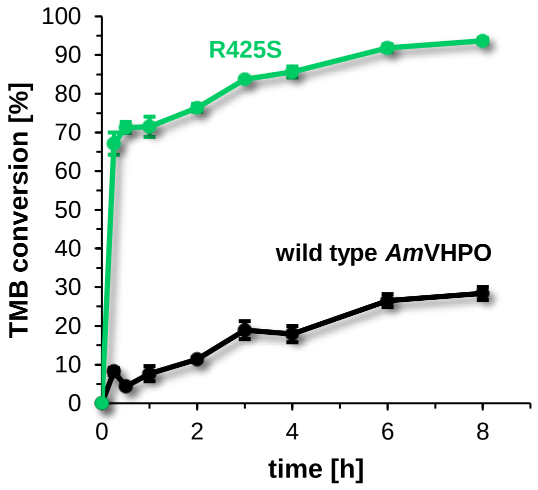

SFigure 8 | TMB (**7**) conversion by chlorination. Black: wild-type*Am*VHPO, green: R425S-*Am*VHPO. Substrate conversion was determined by LC-MS analysis. Data are presented as mean ± SD for n = 3 independent experiments. Source data are provided as a Source Data file. *Am*VHPO = vanadium-dependent haloperoxidase from *Acaryochloris marina*.

4. ENZYME ACTIVITY OF DOUBLE MUTANTS

4.1 Monochlorodimedone assay

All reactions were performed in triplicate using 96-well microplates (Brand® F-bottom, UV-transparent, pureGrade™). A reaction mixture containing MES buffer pH 6 (50 mM), KCl (200 mM), Na_3VO_4 (1 µM), and the respective *Am*VHPO variant (4.4 µg/mL) was prepared, and MCD (50 µM; 1 mM stock solution in 2 M NaOAc) added. The volume per well was 270 µL. To start the measurement, 30 µL H_2O_2 (10 mM, final concentration 1 mM) was added last, and the decrease in absorption (290 nm) was monitored over time (30 min) at 30 °C by UV/Vis spectroscopy. Data were analyzed using Microsoft® Excel® 2019 MSO version 16.0.10366.20016 and OriginPro 2019 version 9.6.0.172.

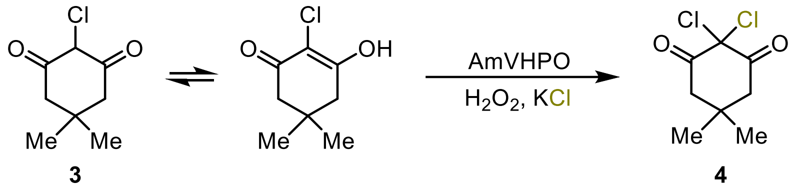

For comparison, the activity of the R425S mutant together with the double mutants R425S, E139G, and R425S, F401G are shown here.

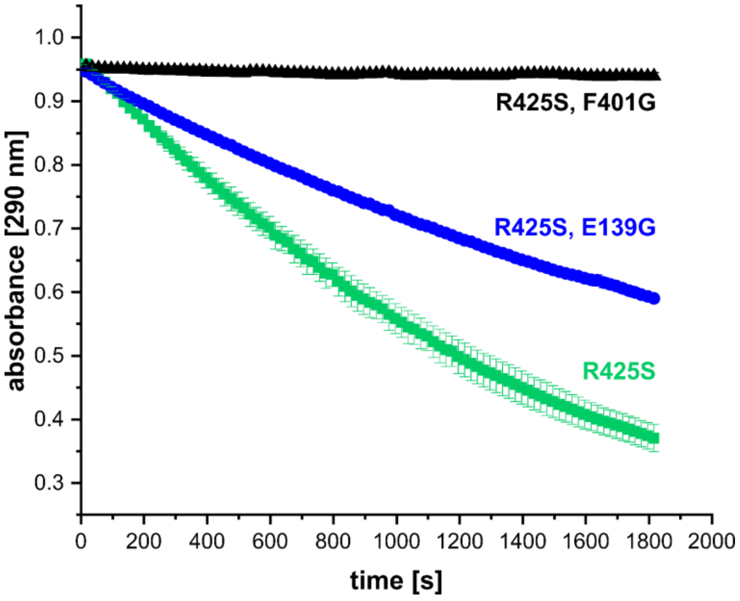

SFigure 9 | Comparison of VHPO variants via MCD assay for chlorination. The mutation of E139 to glycine (blue) leads to slower chlorination of monochlorodimedone, whereas a mutation of F401 to glycine (black) results in a complete loss of chlorination activity. Data are presented as mean ± SD for n = 3 independent experiments. Source data are provided as a Source Data file. MCD = monochlorodimedone; VHPO = vanadium-dependent haloperoxidase.

4.2 Phenol red assay

All reactions were performed in triplicates using 96-well microplates (Brand® F-bottom, pureGrade™). Phenol red (14.3 µM; stock solution prepared in 100 mM NaOH) was added to a mixture of MES buffer pH 6 (50 mM), KX (142 mM, X = Cl, Br), Na_3VO_4 (300 µM) and the respective *Am*VHPO variant (for bromination 6.67 µg/mL; for chlorination 33.3 µg/mL). The reaction was started by the addition of H_2O_2 (10 mM), and the absorption (590 nm) was measured over time (bromination 10 min; chlorination 45 min) at 30 °C. Data were analyzed using Microsoft® Excel® 2019 MSO version 16.0.10366.20016 and OriginPro 2019 version 9.6.0.172.

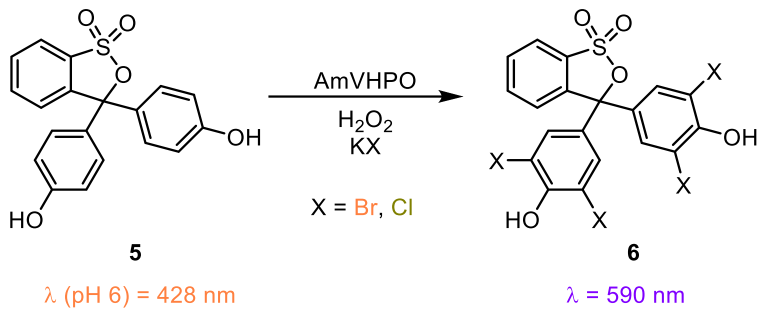

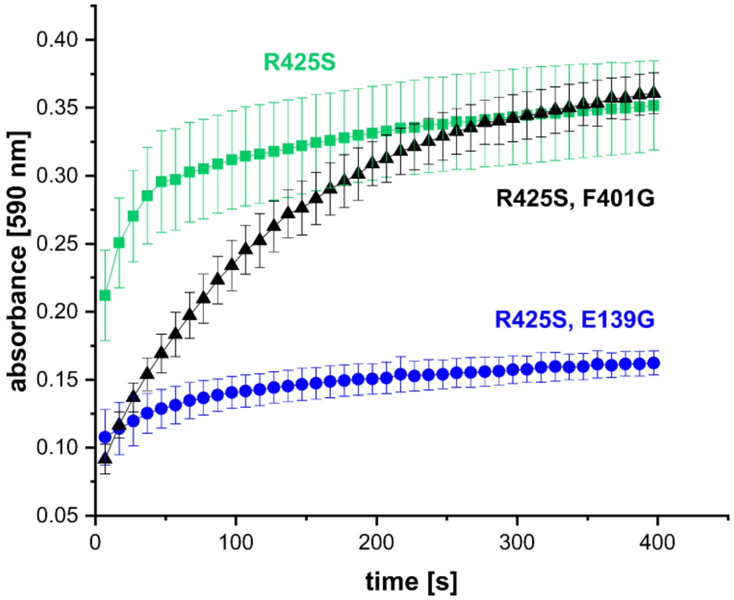

SFigure 10 | Activity assay for the bromination of phenol red. Comparison of the double mutants R425S, E139G, and R425S, F401G for the bromination of phenol red. Mutation of F401 and E139 in the R425S mutants leads to a decreased activity. Data are presented as mean ± SD for n = 3 independent experiments. Source data are provided as a Source Data file.

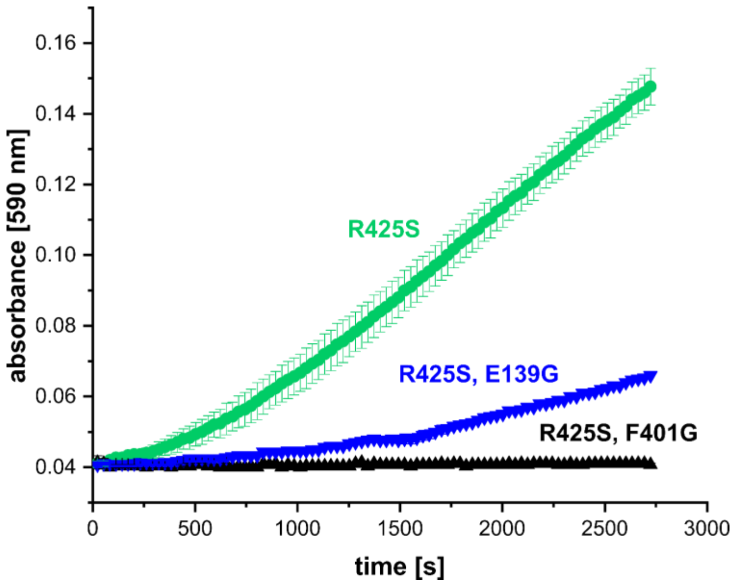

SFigure 11 | Activity assay for the chlorination of phenol red. Mutation of F401 to glycine in the R425S mutant leads to a loss in activity, whereas the substitution of E139 with glycine showed a decreased reaction rate. Data are presented as mean ± SD for n = 3 independent experiments. Source data are provided as a Source Data file.

5. ENZYME KINETICS

A 1 mM stock solution of MCD was prepared in 2 M NaOAc. The 270 µL reaction mixture consisted of MES buffer (50 mM, pH 6.0), MCD (50 µM), KBr (200 mM) or KCl (200 mM), Na_3VO_4 (1 µM), and R425S-*Am*VHPO (final concentration 4.4 µg/mL). For Michaelis-Menten kinetic studies for H_2O_2 consumption, H_2O_2 concentrations were varied in a 2-fold dilution series starting from 10 mM to 0.039 mM (the concentration of the 100 mM stock solution was verified by titration using $KMnO_4$), while keeping KCl concentration at 2 M. Final enzyme concentration for R425S-*Am*VHPO was 4.4 µg/mL. For Michaelis-Menten kinetic chlorination studies, KCl concentrations varied from 50-2000 mM while keeping the H_2O_2 concentration at 1 mM. The final enzyme concentration for R425S-*Am*VHPO was 4.4 µg/mL. For Michaelis-Menten kinetic studies for bromination, KBr concentrations varied from 6.25-1200 µM, keeping the H_2O_2 concentration at 1 mM. The final enzyme concentration for R425S-*Am*VHPO was 2 µg/mL. The decrease in absorbance at 290 nm was monitored continually while keeping the temperature at 30°C and mixing at 410 rpm. Pseudo-first-order kinetics were applied. All assays were performed thrice in triplicates in 96-well polystyrene microplates (Brand® F-bottom, UV-transparent, pureGrade™). The concentration of converted monochlorodimedone was calculated from A_{290} using the following equation.

$$c\,(MCD) = \frac{A_{290}}{\varepsilon_{MCD} \cdot l}$$

$$\varepsilon_{MCD} = 19.9\ mM^{-1}cm^{-1};\ l = 0.91\ cm$$

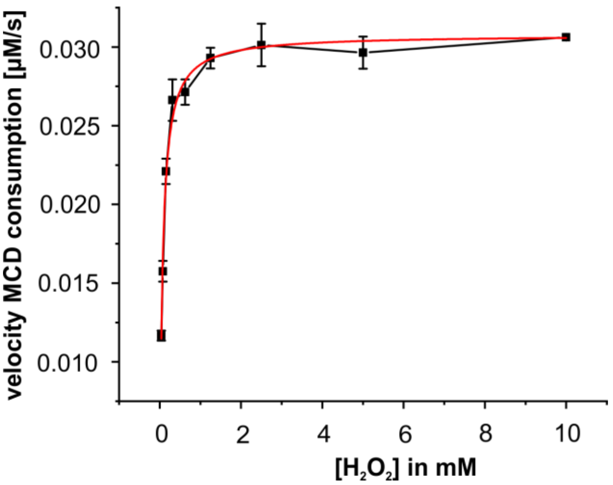

Data were analyzed using Microsoft® Excel® 2019 MSO version 16.0.10366.20016 and OriginPro 2019 version 9.6.0.172.

*Am*VHPO	K_m^{H2O2} [mM]	k_{cat} [s^{-1}]	$k_{cat}^{H2O2}/K_M^{H2O2}$ [s^{-1} mM^{-1}]	A_{spec} [U/mg^{-1}]
wild-type[2]	0.06	n.d.	n.d.	n.d.
R425S	0.066 ± 0.002	0.50 ± 0.03	7.58 ± 1.5	0.41 ± 0.01

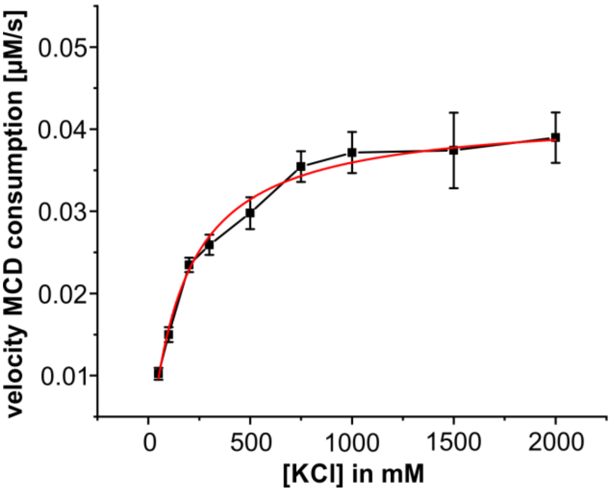

*Am*VHPO	K_M^{Cl} [mM]	k_{cat} [s^{-1}]	k_{cat}^{Cl}/K_M^{Cl} [s^{-1} mM^{-1}]	A_{spec} [U/mg^{-1}]
wild-type[2],[a]	n.d.	n.d.	n.d.	n.d.
R425S	167 ± 12	0.58 ± 0.12	$3.47 \cdot 10^{-3}$	0.47 ± 0.04

Determination of the kinetic parameters was not possible for the wild-type *Am*VHPO because of its low chloroperoxidase activity.

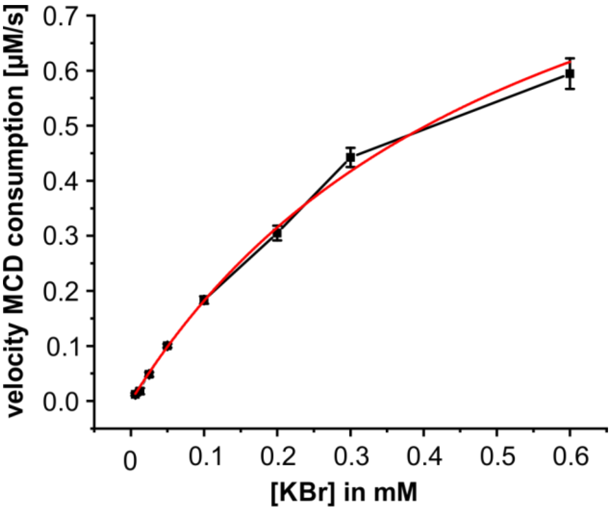

*Am*VHPO	K_M^{Br} [mM]	k_{cat} [s^{-1}]	k_{cat}^{Br}/K_M^{Br} [s^{-1} mM^{-1}]	A_{spec} [U/mg^{-1}]
wild-type[2]	0.4	8.4	20	23.3
R425S	0.55 ± 0.06	46 ± 5	84	43 ± 5

SFigure 12 | Enzyme kinetics of the wild-type *Am*VHPO and its R425S variant. Michaelis-Menten plots of R425S-*Am*VHPO in the MCD assay with varying H_2O_2, chloride, or bromide concentrations. Comparison of Michaelis-Menten constant, turnover number, and specific activity of the wild-type *Am*VHPO. Data are presented as mean ± SD for n = 3 independent experiments. Source data are provided as a Source Data file. *Am*VHPO = vanadium-dependent haloperoxidase from *Acaryochloris marina*; K_M = Michaelis-Menten constant; k_{cat} = catalytic efficiency; A_{spec} = specific activity.

STable 4 | Comparison of the chlorination activity of VHPOs from different organisms and mutants.

Organism	Species	Mutation	K_M^{Cl} [mM]
cyanobacteria	*Acaryochloris marina*	R425S	167
red seaweed	*Corallina officinalis*	wild-type	n.d.
	Corallina pilulifera	wild-type	n.d.
		R397W[3]	780
		R397F[3]	670
brown algae	*Ascophyllum nodosum*	wild-type[4]	344
fungi	*Curvularia inaequalis*	wild-type[5]	0.25

VHPO = vanadium-dependent haloperoxidase; K_M = Michaelis-Menten constant.

6. COMPETITION ASSAY FOR CHLORINATION OF MCD (3) AND TMB (7)

A 2 mM MCD (3) stock solution in 2 M NaOAc and a 2 mM TMB (7) stock solution in DMSO (Acros, 390750010) was prepared. Into 875 µL of a master mix containing MES buffer (50 mM, pH 6.0), KBr (200 mM, for wt_*Am*VHPO) or KCl (200 mM, for R425S_*Am*VHPO), Na_3VO_4 (1 µM), and the respective enzyme (final concentration 4.4 µg mL^{-1}), 100 µL of a TMB (**7**) solution in DMSO was added and incubated for 30 min at rt. Initial concentrations of TMB (**7**) solutions in DMSO were 2 mM, 1 mM, 0.75 mM, 0.5 mM, 0.25 mM, 0.125 mM, and 0 mM (pure DMSO). After incubation, 25 µL of the MCD (**3**) stock solution was added. 270 µL of this solution (triplicates) were pipetted into a 96-well polystyrene microplate (Brand® F-bottom, UV-transparent, pureGrade™), and the reaction was started by the addition of 30 µL 10 mM H_2O_2. The decrease in absorbance at 290 nm was monitored continually while keeping the temperature at 30°C and mixing at 410 rpm. All assays were performed at least three times in triplicates. Pseudo-first-order kinetics were applied. Initial velocities of MCD consumption in the presence of TMB (**7**) were plotted against the ratio of TMB (**7**) to the sum of MCD (**3**) and TMB (**7**). Data were analyzed using Microsoft® Excel® 2019 MSO version 16.0.10366.20016 and OriginPro 2019 version 9.6.0.172.

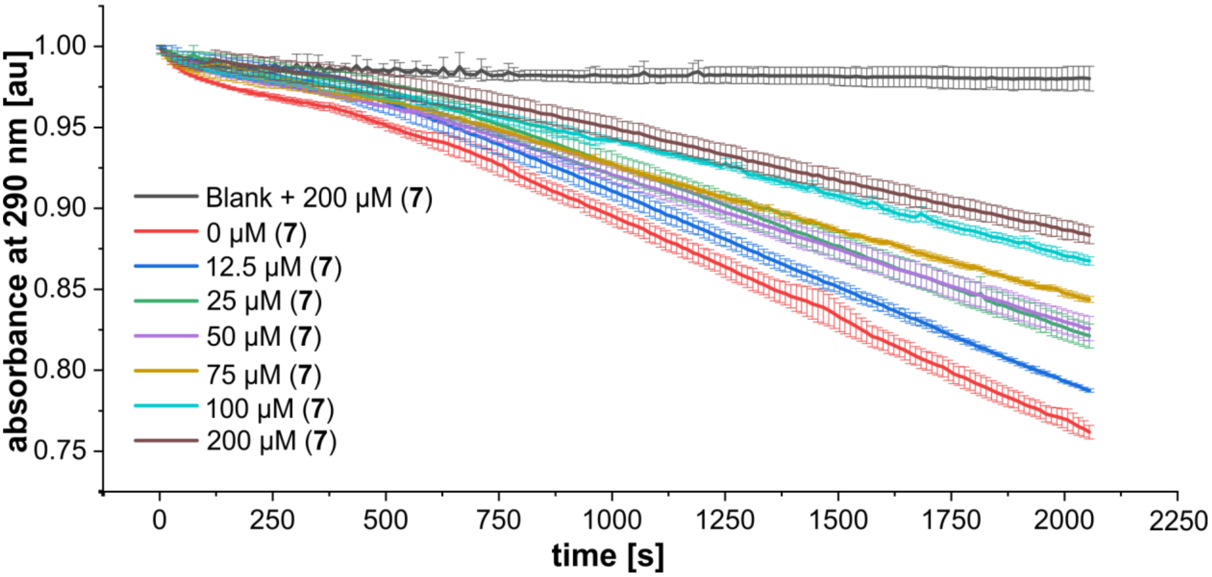

SFigure 13 | Competition of MCD (**3**) consumption by increasing concentrations of TMB (**7**). Initial velocities of MCD (**3**) consumption in the presence of increasing TMB (**7**) concentrations. Data are presented as mean ± SD for n = 3 independent experiments. Source data are provided as a Source Data file. *Am*VHPO = vanadium-dependent haloperoxidase from *Acaryochloris marina*; MCD = monochlorodimedone; TMB = 1,3,5-trimethoxybenzene.

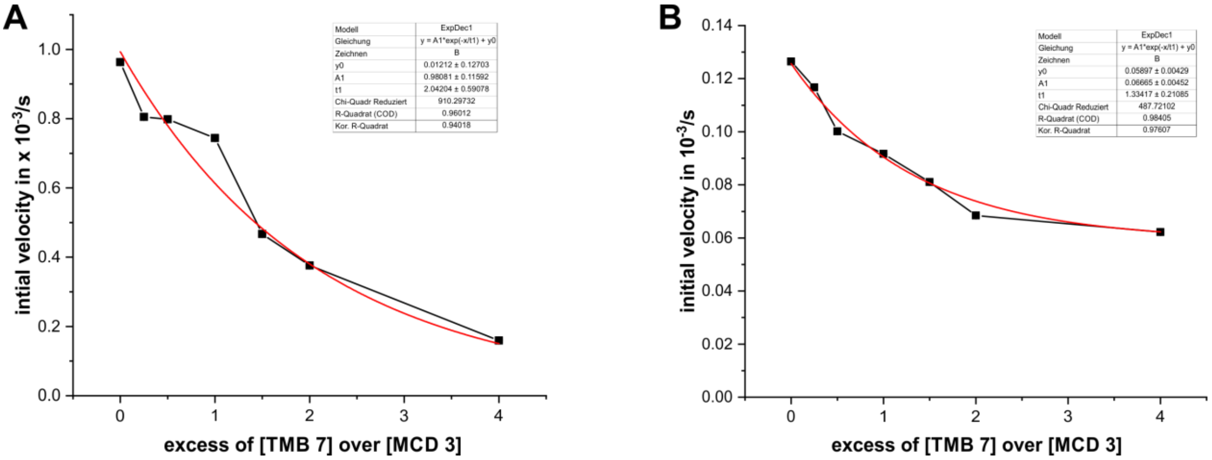

SFigure 14 | Competition of MCD (**3**) consumption with increasing concentrations of TMB (**7**). Plot of initial velocities of MCD (**3**) consumption against the excess of TMB (**7**) over MCD (**3**). A: wild-type *Am*VHPO in the presence of bromide. B: R425S-*Am*VHPO in the presence of chloride. Assays were performed in triplicates. Data are presented as mean ± SD for n = 3 independent experiments. Source data are provided as a Source Data file. *Am*VHPO = vanadium-dependent haloperoxidase from *Acaryochloris marina*; MCD = monochlorodimedone; TMB = 1,3,5-trimethoxybenzene.

SFigure 14 shows the kinetic analysis of the competition assays between MCD (**3**) and TMB (**7**) bromination and chlorination. Shown are the initial velocities of MCD (**3**) halogenation plotted against the excess of the concentrations of TMB (**7**) over MCD (**3**). Both curves follow an exponential decay described by $v = Ae^{-x/\tau} + v_0$. v_0. This describes the velocity the system evolves to at an ever-increasing excess of TMB (**7**). τ describes the excess of TMB (**7**) at which the system reaches a velocity of 1/e (appr. 37%) of the highest velocity. For the graph in SFigure 14A, which shows bromination in the presence of wild-type *Am*VHPO, v_0 eventually approaches 0 with τ at approximately 2, meaning 37% of the starting velocity is reached at a twofold excess of TMB (**7**) over MCD (**3**).

In contrast, in SFigure 14B, where chlorination in the presence of R425S-*Am*VHPO is depicted, the graph plateaus already at a higher level, taking into consideration that chlorination generally proceeds much slower than bromination and initial velocities are therefore lower. Furthermore, τ is at approximately 1.3, meaning TMB (**7**) reduces the initial velocity to 37%, already at a 1.3fold excess over MCD (**3**).

These results lead to two conclusions: The lower τ value in SFigure 14B hints that TMB (**7**) is converted to the chlorinated species quicker compared to the bromination in the wild-type enzyme. If conversion to the chlorinated species is only dependent on the presence of a free-diffusing HOX species interacting with free-diffusing substrates, one would assume that τ is comparable to or higher than the value for bromination, as TMB (**7**) is less reactive than MCD (**3**). However, the lower τ can be attributed to the fact that TMB (**7**) is binding to the mutant enzyme and blocks chlorination of MCD (**3**) already at lower concentrations. Another

interesting observation is that TMB (**7**) cannot reduce the velocity of MCD (**3**) chlorination to zero as opposed to bromination in the wild-type enzyme, meaning that there is a saturation of the effect starting at an approximately 2fold excess of TMB (**7**) over MCD (**3**). Above that value, we do not see a deceleration of MCD (**3**) chlorination, meaning that free-floating, non-binding TMB (**7**) is not converted to the chlorinated species. This underlines our hypothesis that chlorination of TMB (**7**) can only happen in close proximity to the active site of the enzyme. A substrate binding pocket, as we see it in our crystal structure, seems to be a plausible explanation. As TMB (**7**) most probably does not bind with high affinity as it is a small molecule with only one aromatic moiety, it is not surprising that chlorination of MCD (**3**) cannot be completely inhibited in such a case.

7. COMPUTATIONAL SCREENING FOR MUTATION SITES CLOSE TO THE VANADATE BINDING SITE

7.1 Structure preparation

Due to the lack of resolution of the loop at positions 389 to 405 in the PDB, 5LPC crystal structure of the bromoperoxidase[2] AlphaFold2[6] was used to create a model of the monomer structure. Complex assembly was performed with the Rosetta suite of biomolecular modeling software[7] (v.3.13 2021.16+release). A symmetry definition file was created[8] based on the biological assembly of PDB: 5LPC and used to construct a dodecamer consisting of twelve copies of the AlphaFold2 model. The model was minimized with symmetry definitions to resolve potential clashes in the structure.

7.2 Sequence design

The sequence was designed step by step with the deep learning-based method ProteinMPNN.[9] First, the designable space was reduced. For this, the distance to the catalytic residue K228 (max 10 Å) as well as the solvent-accessible surface area (SASA > 0) were used as filters to select positions for the design. Residue distances and SASA were calculated with the Biopython library,[10] the latter using the ShrakeRupley[11] class. Sequence design was performed position by position, keeping all other residue identities fixed. Conditional probabilities were predicted for the predicted monomer and composite dodecamer structure and the 5LPC crystal structure with a temperature of 0.3.

7.3 ΔΔG calculations

Structure optimization and energy calculations were performed in the Rosetta suite. ΔΔGs were computed following the Rosetta implemented cartesian ΔΔG protocol.[12] The dodecamer structure was relaxed in cartesian space, and the ΔΔG for every amino acid substitution was calculated for the 5 best scoring structures out of 500. For each structure and mutation, 10 ΔΔG iterations were performed. The best-scoring mutant out of 10 was used for energy and SASA calculations.

7.4 Visualization

Structural visualizations were created with chimerax.[13]

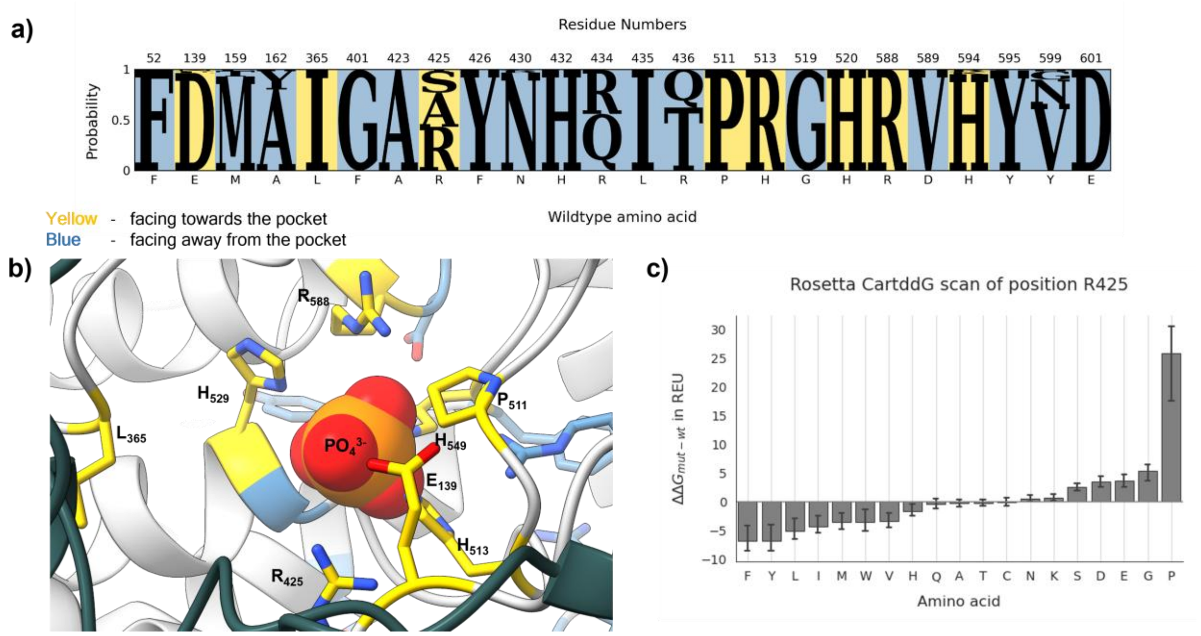

SFigure 15 | Sequence design of residues near the enzymatic pocket. a) Conditional probabilities of amino acids at selected positions were calculated with ProteinMPNN.[9] The designed positions had to be no further than 10 Å away from K428 and a SASA (solvent-accessible surface area)> 0. Positions were furthermore sorted by facing towards (yellow) or away (blue) from the binding pocket. Noteworthy is that position 425 allows amino acids with different sizes and functional groups. **b)** Visualisation of the selected positions in the AlphaFold2 model. Two chains of the homo-dodecamer are shown, and the position of the PO_4^- was superimposed from 5LPC.[2] C) Computed ΔΔG values for each amino acid substitution at position 425 in Rosetta energy units (REU).

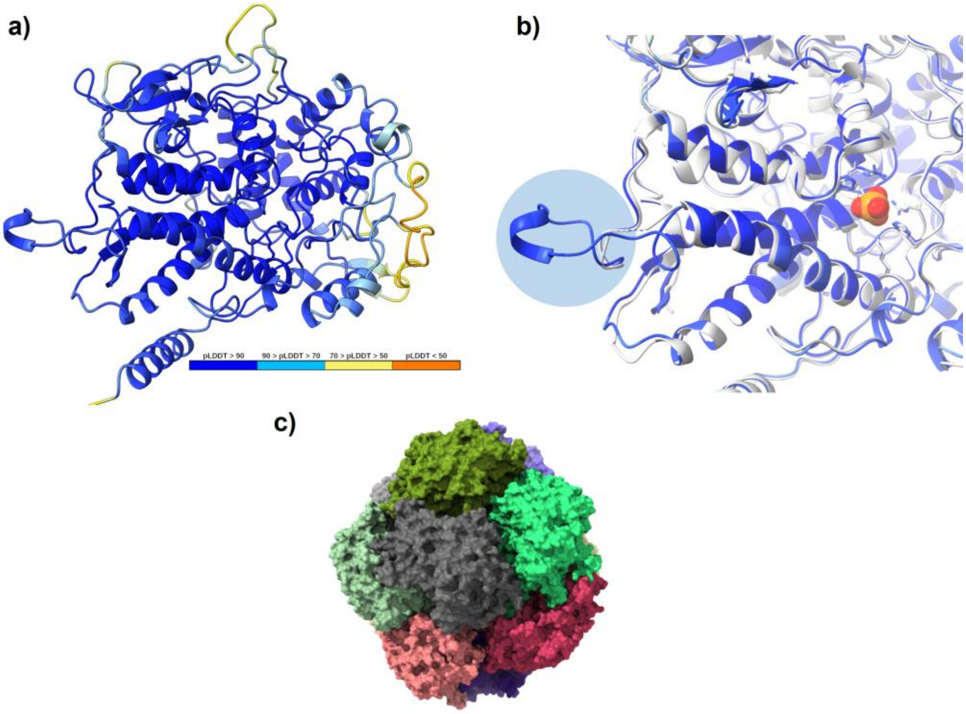

SFigure 16 | AlphaFold model of the vanadium-dependent haloperoxidase from *A. marina.* a) Monomer model colored by confidence score (dark blue – high, orange – low confidence). **b)** Superimposition of the model with the 5LPC crystal structure (white). In 5LPC, the missing loop is highlighted. **c)** The assembled dodecamer structure is shown in surface representation.

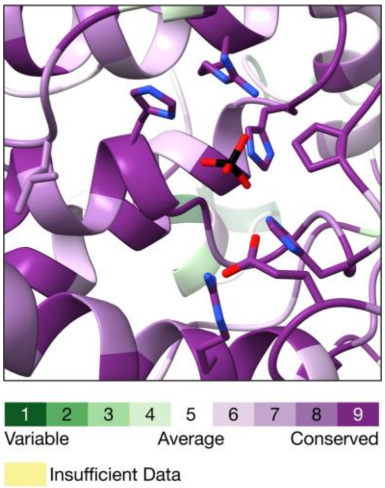

SFigure 17 | In the enzymatic pocket, the conservation scores of selected residues (SFigure13a). All selected residues are highly conserved.

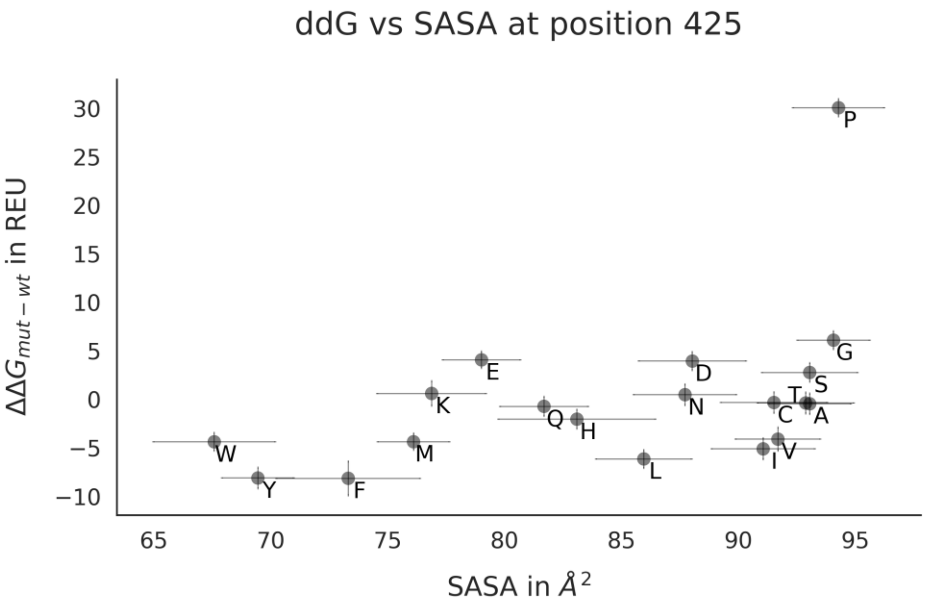

SFigure 18 | ΔΔG at position 425 plotted against the solvent-accessible surface area (SASA) of the catalytic pocket. Large hydrophobic amino acids (W, Y, F) seem to score well but close off the binding pocket. Small amino acids might not have a good Rosetta energy score but increase the solvent accessibility of the pocket. ddG = ΔΔG.

7.5 Molecular dynamics (MD) simulations

To explore the conformational landscape of the loop region spanning position 389 to 405 in the wild type compared to the R425S variant, Hamiltonian replica exchange (HREX) molecular dynamics (MD) simulations were conducted. The starting configurations of wild-type and variant were based on the R425S crystal structure. For the wild-type, the S425 residue was reverse-mutated to Arginine. Parameters for the Histidine-Vanadate complex were adapted from the literature.[14] HREX MD simulations were performed based on the protocol described in Schwarten et. al.[15] Simulation setup and production runs were carried out with GROMACS 2023.1.[16] Both the wild type and the R425S variant systems were modeled as homo-dimers, including the active center and substrate tunnel formed by the two monomers. Each system was solvated in a square water box with a side length of 127.3 Å. Na^+ and Cl^- ions were added to neutralize the system at a salt concentration of ~200 µM. The AMBER99SB-ILDN force field[17] was used for molecular interactions, and the TIP3P[18] water model for solvation. In total, the system contained 62257 water molecules, 232 Na^+ and 190 Cl^- ions in addition to the protein chains.

Minimization was carried out using a steepest descent protocol. Subsequently, two equilibration steps were performed: an equilibration under constant volume (NVT) and a second under constant pressure (NPT) at 300 K, with durations of 1 ns each, employing the v-rescale thermostat[19] and c-rescale barostat.[20] The same thermostat and barostat were employed during all production runs. Eight HREX replicas were carried out, with temperatures spanning from 300 K to 457 K. The “hot” region included all atoms of residues Phe389 to Gly405. Each replica was simulated for 200 ns at constant pressure, with an average exchange rate of 0.36 to 0.41. This resulted in a cumulative trajectory time of 1.6 µs per system.

Principle component analysis was applied to pairwise Cα-distance matrices to characterize the diversity of the conformational landscape across the two simulations Loop conformations were clustered using the DBScan algorithm implemented in the scikit-learn package.[21]

Setup and simulation data are available at https://github.com/ClaraTSchoeder/schoederlab.

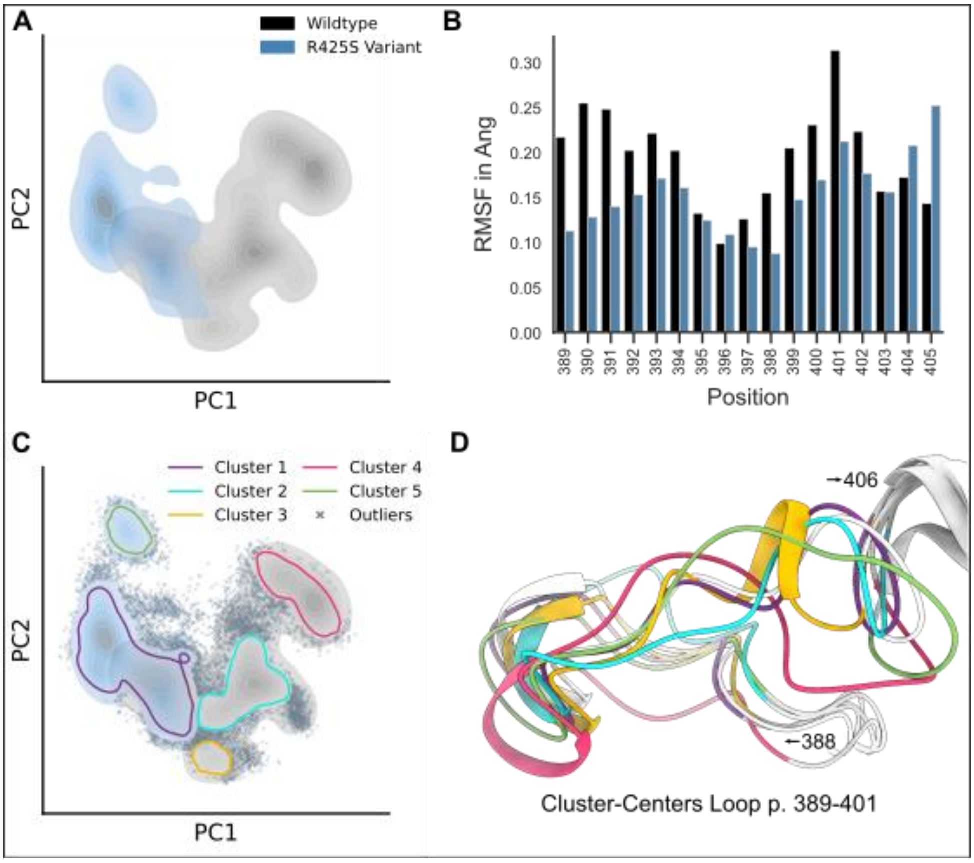

SFigure 19 | Enhanced molecular dynamics simulations of the loop near the active site (position 389 to 401) with structures of the *Am*VHPO R425S variant and wild-type sequence. (A) Principal component analysis (PCA) of the pairwise C-alpha distances of the loop. (B) Root mean square fluctuation (RMSF) of each C-alpha atom in the loop region. (C) DBSCAN clustering of the PCA output. (D) Structure representatives of each cluster colored in the corresponding cluster color. The loop of the R425S variant crystal structure is not colored. PC = principal component; Ang = Angström; DBSCAN = Density-Based Spatial Clustering of Applications with Noise

During the simulation the wildtype and variant structure partly occupy the same but also different loop conformations. Consequently, there is a difference between the loop dynamics in the R425S variant vs the wildtype (SFigure 19A). At the start of the loop (p. 390/391) as well as at the end of the loop (p. 401), the RMSF of the wild-type is increased compared to the variant. This might indicate a reduced stability of the loop in the wild type (SFigure 19B). These positions also agree with the missing resolution in the loop in the wild-type crystal structure. Even though the variant also shows flexibility, the respective cluster of a different conformation (Cluster 5) is rarely occupied (SFigure 19C). On the other side, cluster 4 is highly occupied in the wild-type simulations, which might indicate a fluctuation between this conformation and the conformations in cluster 2 (SFigure 19C). The conformations in cluster 4 highly deviate from the crystal structure, which can be seen in SFigure 19D.

8. CRYSTALLIZATION

8.1 Crystallization of *Am*VHPO variants

*Am*VHPO (15 mg/mL) was crystallized by the hanging drop vapor diffusion method at 20 °C. For co-crystallization experiments, the surrogate 1,3,5-trimethoxybenzene (TMB, **7**, 100 mM stock solution in acetonitrile) was added to *Am*VHPO variants in a final concentration of 1 mM. Crystallization drops had a maximum volume of 2 µl with a 2:1 ratio of protein and reservoir solution (0.1 M Tris pH 8.5, 2 M $NH_4H_2PO_4$ (Sigma, 2004005), 0.5 mM K_3VO_4, FisherScientific, 11328308). Crystals were cryoprotected by a 7:3 mixture of mother liquor and 100% (v/v) ethylene glycol (Sigma, 293237) and subsequently vitrified in liquid nitrogen.

8.2 Structure determination of *Am*VHPO variants

Datasets of *Am*VHPO variants were recorded with synchrotron radiation of λ = 1.0 Å at the beamline X06SA, Swiss Light Source (SLS), Paul Scherrer Institute, Villigen, Switzerland. Reflection intensities were evaluated with the program package XDS, and data reductions were carried out with XSCALE[22] (Table S5). Each structure was solved by Patterson search calculations with REFMAC5[23] and coordinates of *Am*VHPO (PDB ID 5LPC).[2] The models were completed using COOT[24] in combination with REFMAC5, respectively. Notably, the *Am*VHPO_R425S was determined in the absence and presence of 1,3,5-trimethoxybenzene (TMB, **7**). Restrained and TLS (Translation/Libration/Screw) REFMAC refinements yielded excellent R_{work} and R_{free} values as well as root-mean-square deviation (rmsd) values of bond lengths and angles. All crystal structures have been deposited in the RCSB Protein Data Bank (Table S5).

STable 5 | Crystallographic data collection and refinement statistics

	*Am*VHPO_R425D	*Am*VHPO_R425S	*Am*VHPO_R425S: TMB (7)
**Crystal parameters**			
Space group	$F4_132$	$F4_132$	$F4_132$
Cell constants	a=308.4 Å	a=306.7 Å	a=305.8 Å
LplA1 / AU[a]	1	1	1
**Data collection**			
Beamline	X06SA, SLS	X06SA, SLS	X06SA, SLS
Wavelength (Å)	1.0	1.0	1.0
Resolution range (Å)[b]	30-3.5 (3.6-3.5)	30-3.3 (3.4-3.3)	30-3.3 (3.4-3.3)
No. observations	80007	92577	81200
No. unique reflections[c]	15830	17667	17934
Redundancy	5.0	5.2	4.5
Completeness (%)[b]	96.5 (96.6)	92.3 (94.8)	94.3 (95.5)
R_{merge} (%)[b, d]	12.2 (63.2)	11.9 (64.1)	10.8 (62.9)
I/σ (I)[b]	9.4 (2.4)	9.9 (2.3)	9.6 (2.0)
**Refinement (REFMAC5)**			
Resolution range (Å)	30-3.5	30-3.3	30-3.3
No. refl. working set	15012	16761	17015
No. refl. test set	790	882	895
No. non hydrogen	4886	4937	4950
No. of ions	2	2	1
No. of ligands	0	0	1
Solvent	0	0	0
R_{work}/R_{free} (%)[e]	22.4 / 24.4	21.0 / 23.3	21.6 / 24.8
r.m.s.d. bond (Å) / (angle)[f]	0.002 / 1.2	0.002 / 1.2	0.002 / 1.2
Average B-factor ($Å^2$)	96.5	81.2	83.8
Ramachandran Plot (%)[g]	96.1 / 3.9 / 0	96.2 / 3.8 / 0	96.7 / 3.3 / 0
PDB accession code	**8Q20**	**8Q21**	**8Q22**

[a] Asymmetric unit

[b] Values in parentheses for resolution range, completeness, R_{merge}, and I/σ (I) correspond to the highest resolution shell

[c] Data reduction was carried out with XDS and from a single crystal. Friedel pairs were treated as identical reflections

[d] $R_{merge}(I) = \Sigma_{hkl}\Sigma_j \mid I(hkl)_j - \langle I(hkl)\rangle \mid / \Sigma_{hkl} \Sigma_j I(hkl)_j$, where $I(hkl)_j$ is the jth measurement of the intensity of reflection hkl and <I(hkl)> is the average intensity

[e] $R = \Sigma_{hkl} \mid |F_{obs}| - |F_{calc}| \mid / \Sigma_{hkl} |F_{obs}|$, where R_{free} is calculated without a sigma cut-off for a randomly chosen 5% of reflections, which were not used for structure refinement, and R_{work} is calculated for the remaining reflections

[f] Deviations from ideal bond lengths/angles

[g] Percentage of residues in favored region / allowed region / outlier region

8.3 Electron density maps

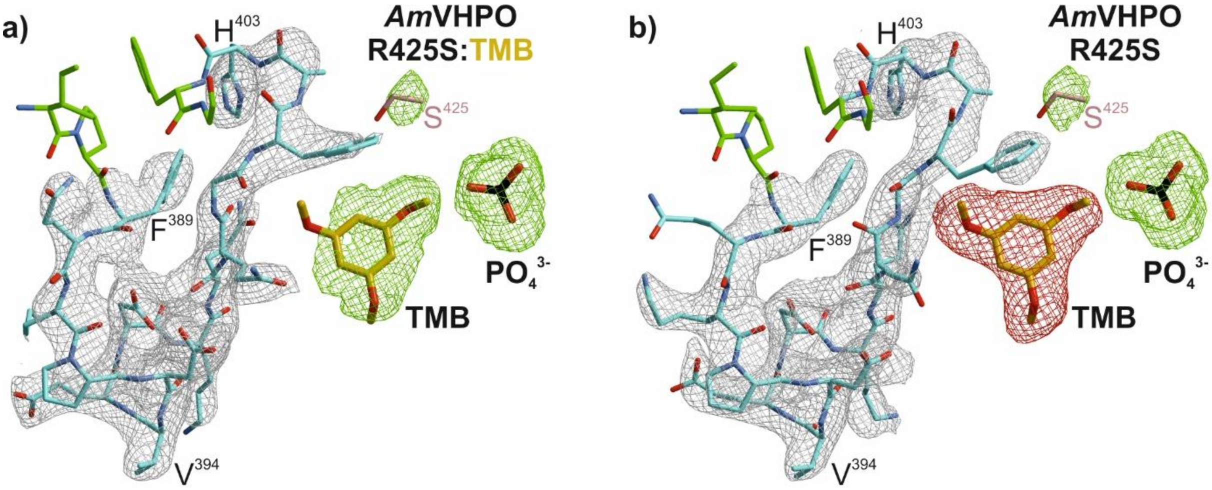

SFigure 20 | Electron density maps of *Am*VHPO-R425S mutant. a) In the R425S:TMB complex (color-coding according to Figure 5, PDB ID 8Q22), the region between residue 389 and 403 is shown in the 2F_O-F_C electron density map (grey mash, contoured to 1.0 σ). TMB (**7**), phosphate, and Ser425 have been omitted for phasing. The corresponding F_O-F_C electron density map contoured to +2.5 σ (green) highlights both ligands and the mutation site. **b)** In the R425S apo structure (PDB ID 8Q21), TMB (**7**) was modeled with coordinates of the R425S:TMB complex. For phasing, the temperature factor of TMB (**7**) was set to the average for protein residues (81 Å^2, STable 5), while phosphate and Ser425 have been omitted. The F_O-F_C electron density map contoured to -2.5 σ (red) proves the absence of TMB (**7**). *Am*VHPO = vanadium-dependent haloperoxidase from *Acaryochloris marina*; TMB = 1,3,5-trimethoxybenzene.

8.4 Comparison of the possible loop regions in the *Am*VHPO variants

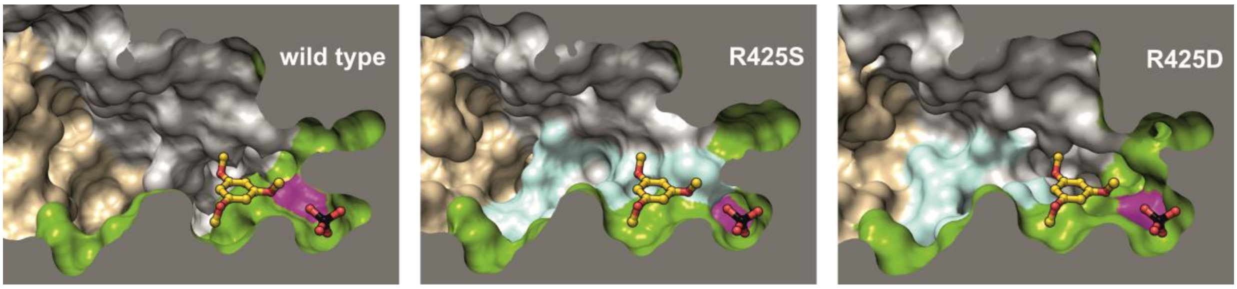

SFigure 21 | Surface cross-section of *Am*VHPO variants in complex with 1,3,5-trimethoxybenzene (7). The cartoon represents one of 12 active sites in *Am*VHPO. The substrate binding pocket comprises two *Am*VHPO subunits shown in green and grey, respectively. Other subunits of the dodecamer are colored brown. TMB (**7**) is only bound in the R425S mutant (PDB ID 8Q22). Superposition of the liganded *Am*VHPO-R425S mutant with wild-type (PDB ID 5LPC) and R425D (PDB ID 8Q20) structures illustrates how **7** may bind in these variants. Residue 425 (magenta) has a significant impact on the shape of the specificity pocket: TMB (**7**) is stabilized by a defined loop region from the adjacent subunit (residues 390-404, highlighted in cyan), which is fully resolved in R425S, flexible in the wild-type structure and partially present in R425D. *Am*VHPO = vanadium-dependent haloperoxidase from *Acaryochloris marina*; TMB = 1,3,5-trimethoxybenzene.

9. COMPARISON OF THE STRUCTURE AND THE CHLORINATION ACTIVITY OF DIFFERENT VHPOS

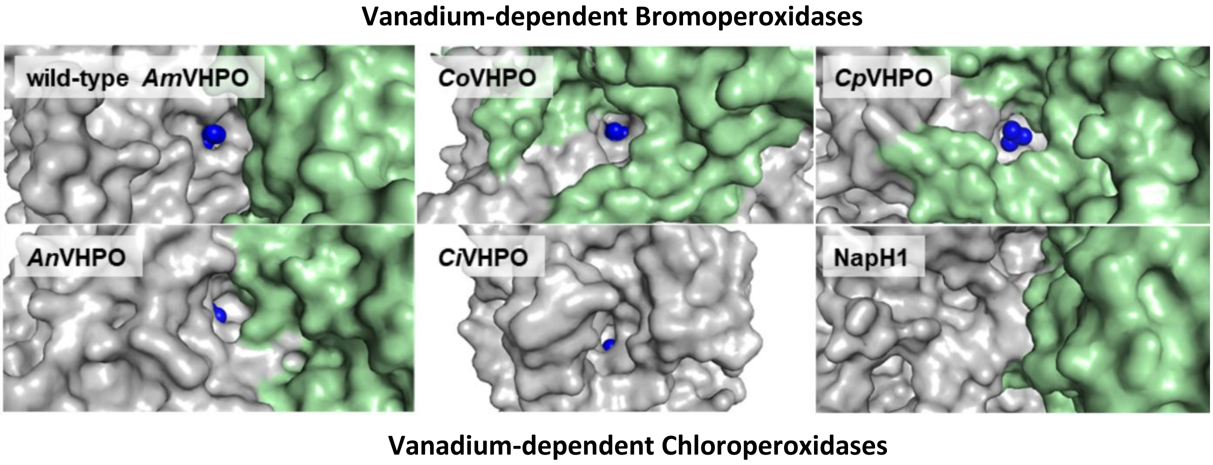

SFigure 22 | Access to the active site in different VHPOs. Enzymes are depicted as dimeric units (grey and light green), except *Ci*VHPO, which mainly acts as a monomer. In the case of the dodecamers wild-type *Am*VHPO, *Co*VHPO, and *Cp*VHPO, a combination of two subunits was chosen that resembles the densest packing around the prosthetic vanadate and phosphate group, respectively (blue spheres), as it can be found in the complete protein. *Am*VHPO = vanadium-dependent haloperoxidase from *Acarychloris marina*; *Co*VHPO = vanadium-dependent haloperoxidase from *Corallina officinalis*; *Cp*VHPO = vanadium-dependent haloperoxidase from *Corallina pilulifera*; *An*VHPO = vanadium-dependent haloperoxidase from *Ascophyllum nodosum*; *Ci*VHPO = vanadium-dependent haloperoxidase from *Curvularia inaequalis*; NapH1 = vanadium-dependent napyradiomycin haloperoxidase from *Streptomyces sp. CNQ-525*.

The upper row in SFig.22 depicts exclusively vanadium-dependent bromoperoxidases. In all three of them, the active site vanadate is located at the end of a broad funnel, ensuring fast substrate access and release of the electrophilic HOBr species for bromination outside the enzyme. The lower row represents vanadium-dependent peroxidases capable of conducting chlorinations. The *An*VHPO shows weak chloroperoxidase activity and is rather communicated as a bromoperoxidase. The active site is solvent-exposed, but dimerization leads to a tunnel-like structure at the interface of the two subunits shielding the prosthetic group. In the strong chloroperoxidase *Ci*VHPO, this group is even more enclosed and buried at the end of a tunnel, in this case, formed in the monomeric structure already (no dimeric structure available). In NapH1, a chloroperoxidase catalyzing stereoselective chlorofunctionalizations, vanadate is no longer solvent exposed and entirely covered by the protein. This qualitative inspection of different haloperoxidases reveals a tendency to chlorination activity when the enzyme environment somewhat shields the prosthetic group.

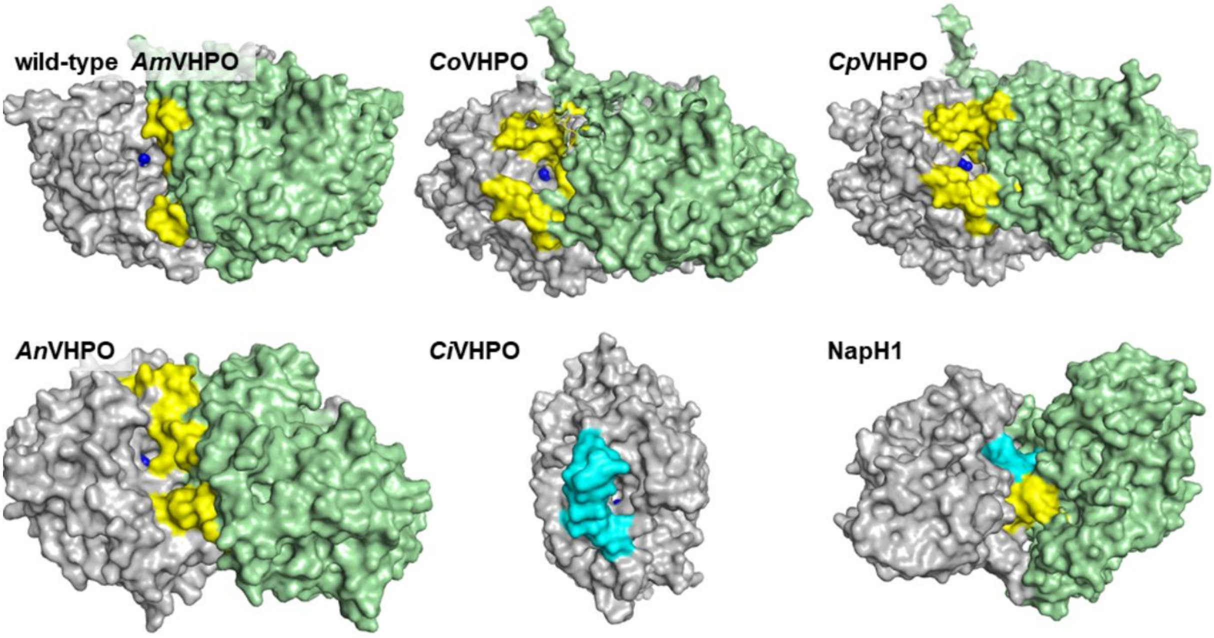

SFigure 23 | Influence of loop structures on formation of the active site entrance. Enzymes are depicted as dimeric units (grey and light green), except *Ci*VHPO, which mainly acts as a monomer. In the case of the dodecamers wild-type *Am*VHPO, *Co*VHPO, and *Cp*VHPO, a combination of two subunits was chosen that resembles the densest packing around the prosthetic vanadate and phosphate group, respectively (blue spheres), as it can be found in the complete protein. Cyan represents a loop in the grey subunit and yellow in the light-green subunit. *Am*VHPO = vanadium-dependent haloperoxidase from *Acarychloris marina*; *Co*VHPO = vanadium-dependent haloperoxidase from *Corallina officinalis*; *Cp*VHPO = vanadium-dependent haloperoxidase from *Corallina pilulifera*; *An*VHPO = vanadium-dependent haloperoxidase from *Ascophyllum nodosum*; *Ci*VHPO = vanadium-dependent haloperoxidase from *Curvularia inaequalis*; NapH1 = vanadium-dependent napyradiomycin haloperoxidase from *Streptomyces sp. CNQ-525*.

In the five dimeric structures, the access to the active site forms close to the interface between two protein subunits, and the interaction between those subunits is primarily determined by loop structures. In fact, in NapH1, there are two prominent loops in the respective *N*-terminal part of each subunit (cyan belonging to the grey subunit, yellow belonging to the light-green subunit) that strongly interact with each other at the monomers' interface and are responsible for the dense packing around the active site. Upon inspection of *Co*VHPO and *Cp*VHPO, which are more related to *Am*VHPO, it becomes apparent that the loop of the neighboring subunit (light green) forms the shape of the active site in the grey subunit. While in the wild-type *Am*VHPO, especially the upper loop is not resolved, leaving broad access to the vanadate group, two distinct loops in *Co*VHPO and *Cp*VHPO frame the active site entrance and form a wide funnel. In *An*VHPO, however, it is mainly the upper loop that engages in tunnel formation in front of the active site, correlating with an onset of chlorination activity. This loop is in the same region as the non-resolved loop in *Am*VHPO that becomes ordered in the R425S mutant, hinting at the influence of this structure on halogen selectivity. The chloroperoxidase *Ci*VHPO,

acting as a monomer, also uses a loop structure in front of the active site that closes off the entrance and takes part in tunnel formation, underlining the importance of defined loop formation at the active site.

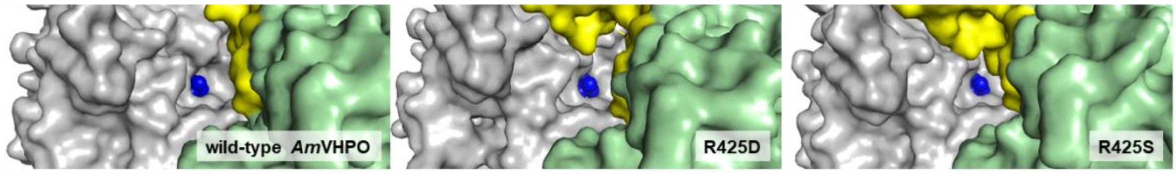

SFigure 24 | Structural comparison of the access to the prosthetic group in the wild-type *Am*VHPO and variants R425D (no chlorination activity) and R425S (chlorination activity). Enzymes are depicted as dimeric units (grey and light green), except *Ci*VHPO, which mainly acts as a monomer. In the case of the dodecamers wild-type *Am*VHPO, *Co*VHPO, and *Cp*VHPO, a combination of two subunits was chosen that resembles the densest packing around the prosthetic vanadate and phosphate group, respectively (blue spheres), as it can be found in the complete protein. Cyan represents a loop in the grey subunit, and yellow is a loop in the light-green subunit. *Am*VHPO = vanadium-dependent haloperoxidase from *Acarychloris marina.*

While the designated loop structure from residues 390 – 404 in the wild-type enzyme is not resolved, pointing to high flexibility, this area becomes more defined in variant R425D and is ultimately resolved in R425S. As seen, the area around the prosthetic group becomes progressively buried in a tunnel structure with increasing loop definition, underlining our hypothesis that this structural change influences halogen selectivity.

Overall, the structural comparison of different VHPOs points toward a correlation between the chloroperoxidase activity and the enclosure of the active site prosthetic group within the enzyme, which is influenced by loops surrounding the entrance to the active site.

10. BROMINATION AND CHLORINATION ACTIVITY OF CORALLINA VHPOS AND THEIR R→S MUTANTS

In order to test if the exchange of Arg i-3 from the active site Lys (i) in other VBPOs shows similar biological effects as in the VBPO of *A. marina*, we chose to substitute R396 and R397 in the bromoperoxidases of *Corallina officinalis* and *Corallina pilulifera*, respectively. Both show a similar three-dimensional structure compared to the *Am*VHPO and also strong sequence similarity. A sequence alignment of the complete amino acid sequence for these VBPOs is shown in SI chapter 12. Here, the mutated region is shown:

```
                          425
A. marina:          ...TRGLKAVRFQKFNV...
                          396
C. officinalis:     ...TRALKAVRYQKFNI...
                          397
C. pilulifera:      ...TRALKAVRYQKFNI...
```

10.1 Monochlorodimedone assay

Assays were performed as described in chapter 4.1 and performed in triplicate, n = 3.

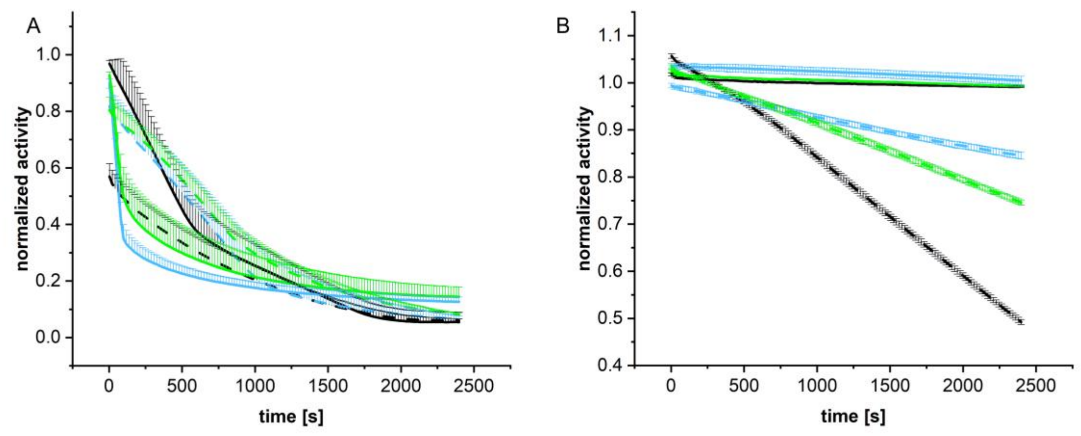

SFigure 25 | Haloperoxidase activity of different VHPOs and respective R→S mutants. A: bromoperoxidase activity; B: chloroperoxidase activity. —— wild-type *Am*VHPO, - - - R425S-*Am*VHPO, —— wild-type *Co*VHPO, - - - R396S-*Co*VHPO, —— wild-type *Cp*VHPO, - - - R397S-*Cp*VHPO. Activity was evaluated using MCD assay in presence of potassium chloride or bromide. Data are presented as mean ± SD for n = 3 independent experiments. Source data are provided as a Source Data file.

All enzymes show bromination activity as shown in SFig. 10A. While for R425S-*Am*VHPO, bromoperoxidase activity slightly increases compared to wild-type *Am*VHPO, R396S-*Co*VHPO and R397S-*Cp*VHPO show a slight decrease. In contrast to that, all mutants show increased chloroperoxidase activity compared to wild-type enzymes (SFig. 10B).

10.2 TMB (7) conversion

The chlorination of TMB (**7**) in the presence of mutants of *Corallina* species was investigated as described in chapter 11.1.

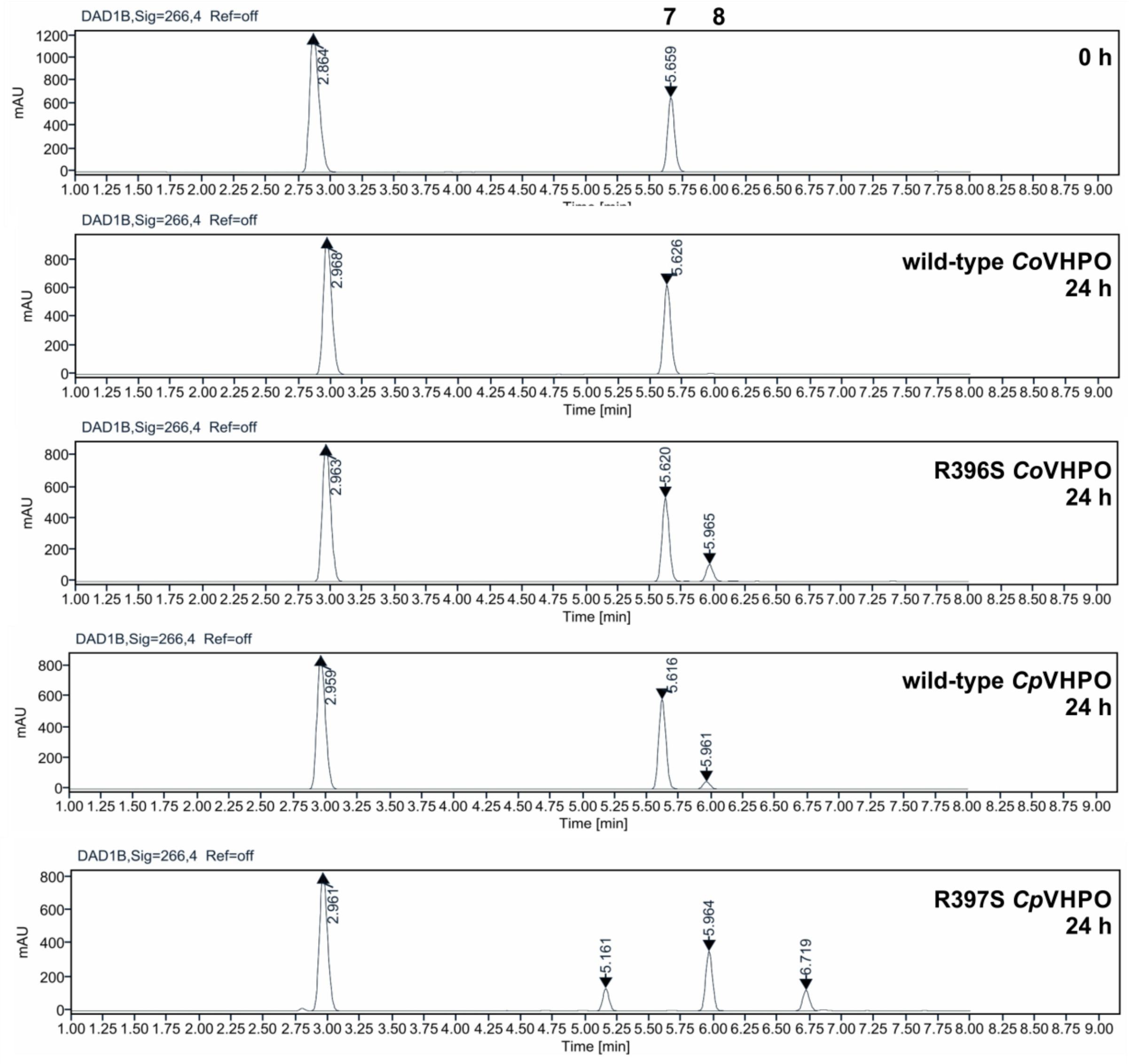

SFigure 26 | Chlorination of TMB (7) by different VHPOs and respective R→S mutants.

All mutants show conversion of TMB (**7**) in the presence of potassium chloride. While for R396S-*Co*VHPO product formation occurred only slowly, R397S-*Cp*VHPO gave complete conversion with mainly formation of chlorinated TMB (**8**), resembling varying activities found in the MCD-assay (chapter 5.1). Similarly to R425S-*Am*VHPO, products at retention times around 5.1 and 6.7 min were found, that did not show any chlorination pattern.

11. AROMATIC CHLORINATION CATALYZED BY R425S-*AM*VHPO

11.1 General Procedure

R425S-*Am*VHPO variant (273.7 µg/mL) was preincubated with a 1.74 mM solution of Na_3VO_4. Substrate (7.43 mM; 11.9 µmol) was solved in an MES (50 mM pH 6) buffered solution of water and acetonitrile (1/1 *v*/*v*), containing KCl (4.4 eq., 32.5 mM) H_2O_2 (16.4 mM) and the enzyme in a total volume of 1.60 mL. The reaction mixture was incubated for 24 h at 30 °C. Afterward, halfmolar amounts of standard with respect to initial substrate concentration were added, and the reaction was stopped by adding a sat. $NaCl/(NH_4)_2SO_4$ solution and extracted with ethyl acetate. Standards were phenol (LC-MS), *n*-dodecanol (Sigma, 443816) (GC-MS) or 1,4-dibromobenzene (abcr, AB111795) (NMR). The solvent was removed under reduced pressure, and the product was analyzed without further purification. ^{1}H-NMR spectra are shown for the purified main products. LC-MS data were obtained using OpenLab CDS Acquisition version 2.6 and OpenLab CDS Analysis version 2.6. GC-MS data were obtained using MassHunter Workstation GC/MS Data Acquisition version 10.2.489 and MassHunter Workstation Qualitative Analysis version 10.0 NMR data were analyzed using MestReNova version 14.1.0-24037.

11.2 Chlorination of 1,3,5-trimethoxybenzene (7)

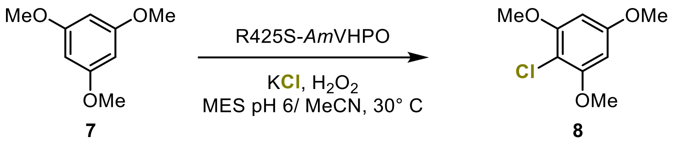

time	Conversion of **7**	Isolated yield of **8**
24 h	75%	59%

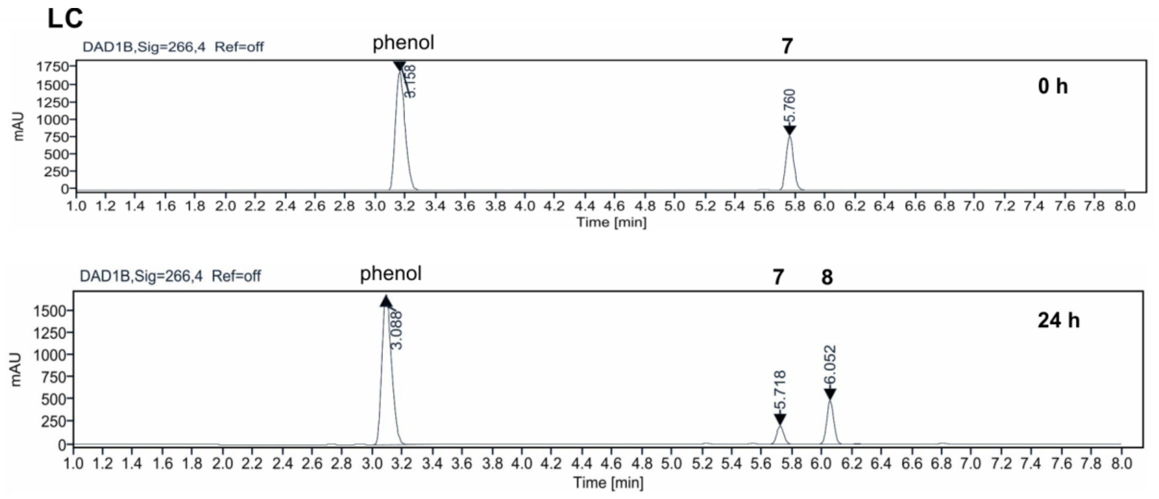

SFigure 27 | Chlorination of TMB (7) using *Am*VHPO-R425S

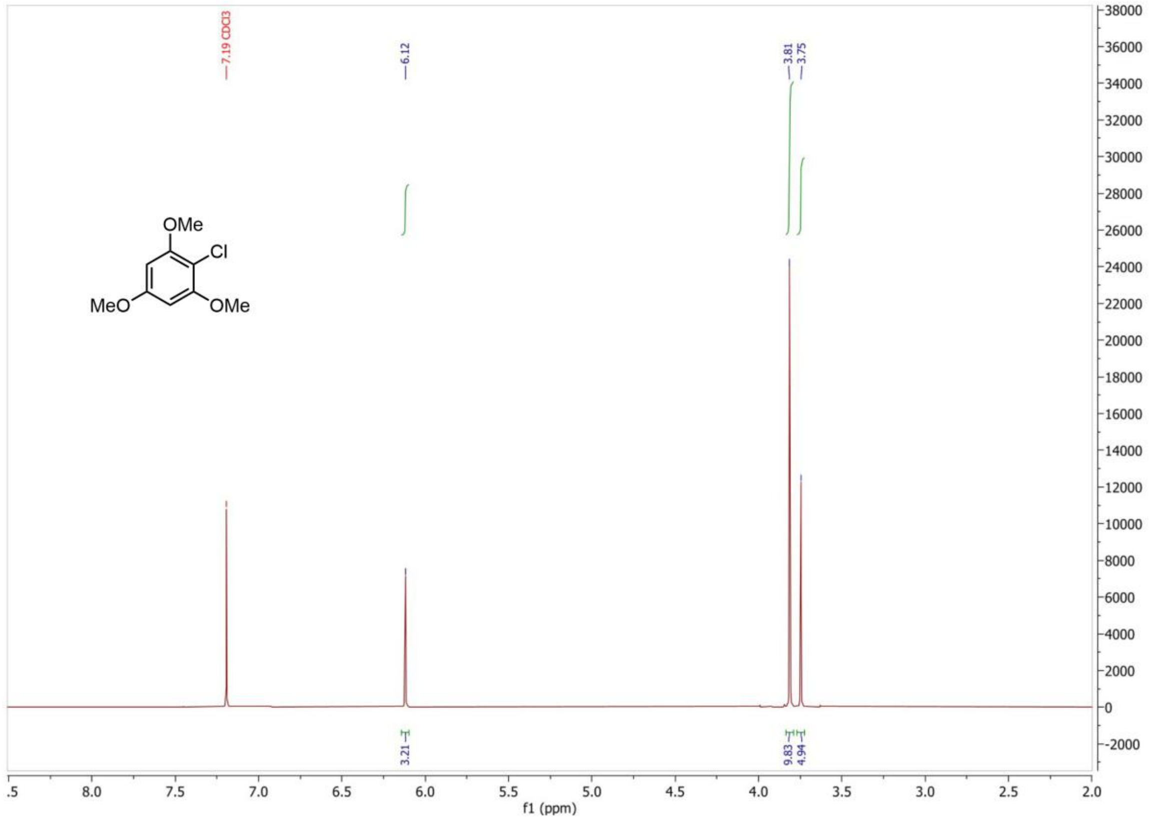

SFigure 28 | ^{1}H-NMR spectrum of product 8

2-Chloro-1,3,5-trimethoxybenzene (8):[2] **^{1}H-NMR** (400 MHz, $CDCl_3$) δ = 6.12 (s, 2H), 3.83 (s, 6H), 3.75 (s, 3H) ppm; **MS** (**8**, EI, 70eV): m/z (%) = 204 $[M]^+$ (12), 202 $[M]^+$ (35), 168 $[M]^+$ (100), 139 $[M]^+$ (92), 125 $[M]^+$ (21), 65 $[M]^+$ (29).

The physical and spectroscopic data agree with those reported in the literature.[2]

11.3 Chlorination of thymol (S1)

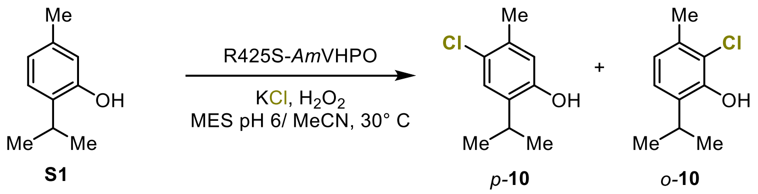

time	Conversion of **S1**	Ratio of *p*-**10**:*o*-**10** (enzymatic conversion)	Isolated yield of **10**	Ratio of *p*-**10**:*o*-**10** (chemical conversion)
24h	66%	100:0	44%	55:45

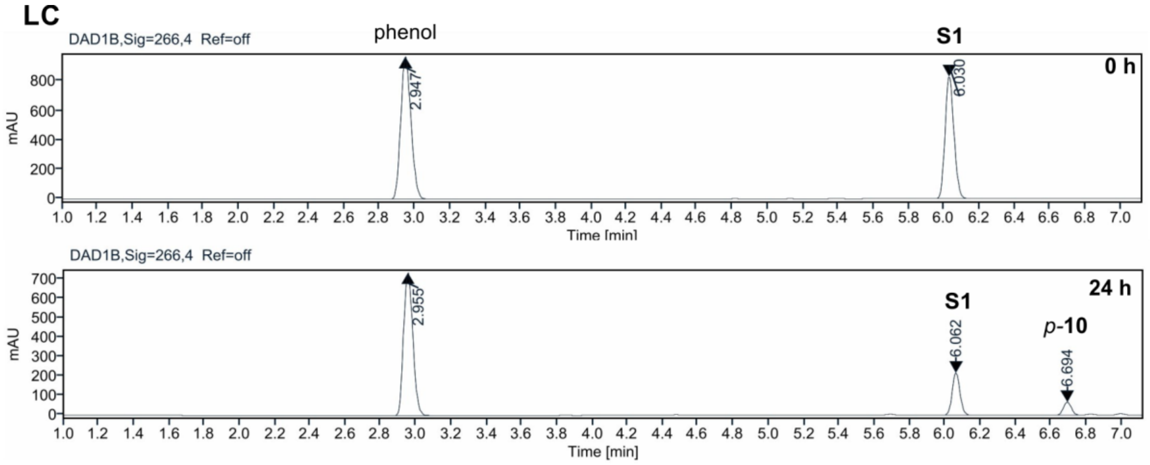

SFigure 29 | Chlorination of thymol (S1) using *Am*VHPO-R425S

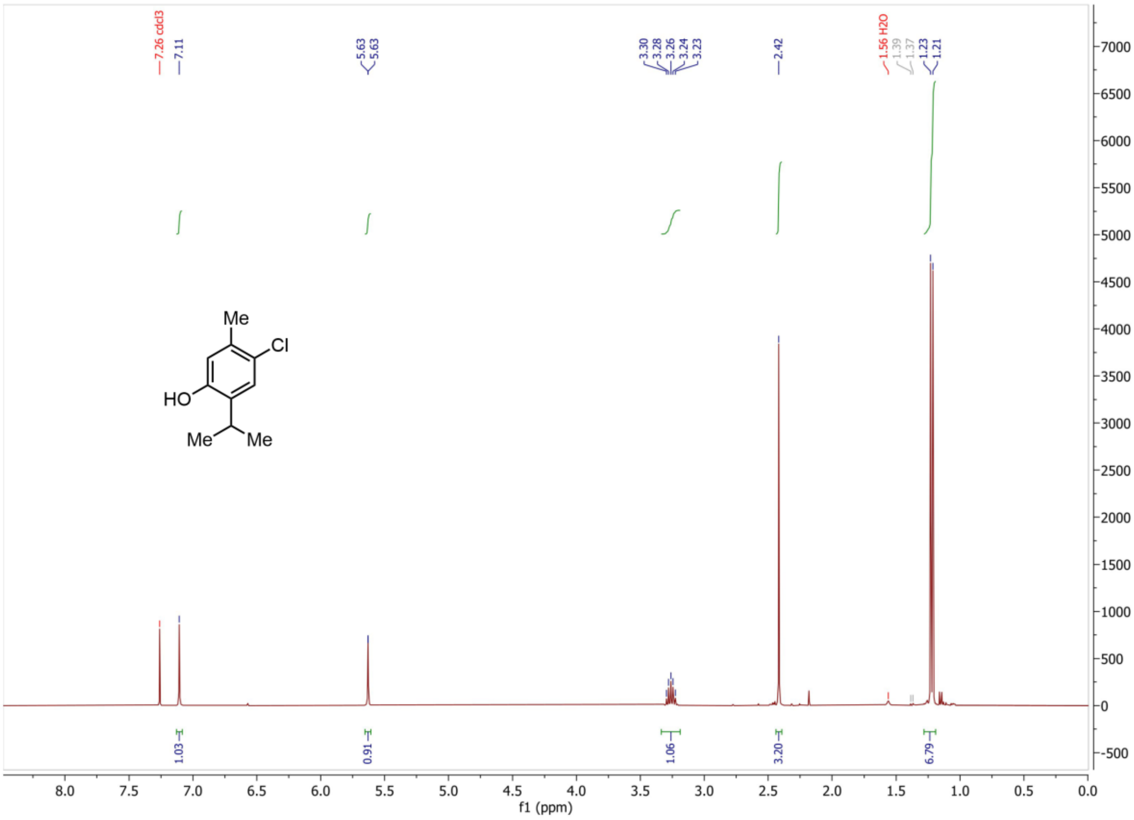

SFigure 30 | ^{1}H-NMR spectrum of product *p*-10

4-Chloro-5-methyl-2-(propan-2-yl)phenol (*p*-10):[2] **^{1}H-NMR** (500 MHz, $CDCl_3$) δ = 7.11 (s, 1H), 5.63 (s, 1H), 3.30-3.23 (m, 1H), 2.42 (s, 3H), 1.22 (d, *J* = 6.9 Hz, 6H) ppm; **MS** (*p*-**10**, EI, 70eV): m/z (%) = 186 $[M]^+$ (8), 184 $[M]^+$ (29), 171 $[M]^+$ (30), 169 $[M]^+$ (100).

The physical and spectroscopic data agree with those reported in the literature.[2]

11.4 Chlorination of 1,3-dimethoxybenzene (S3)

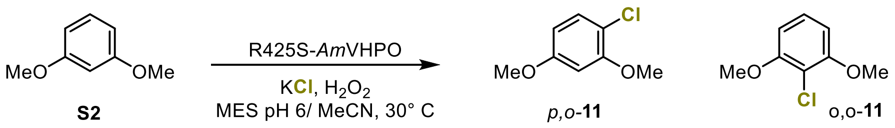

time	Conversion of **S2**	Ratio of *p,o*-**11**:*o,o*-**11** (enzymatic conversion)	Ratio of *p,o*-**11**:*o,o*-**11** (chemical conversion)	Isolated yield of *p,o*-**11**
24 h	89%	99:1	100:0	82%

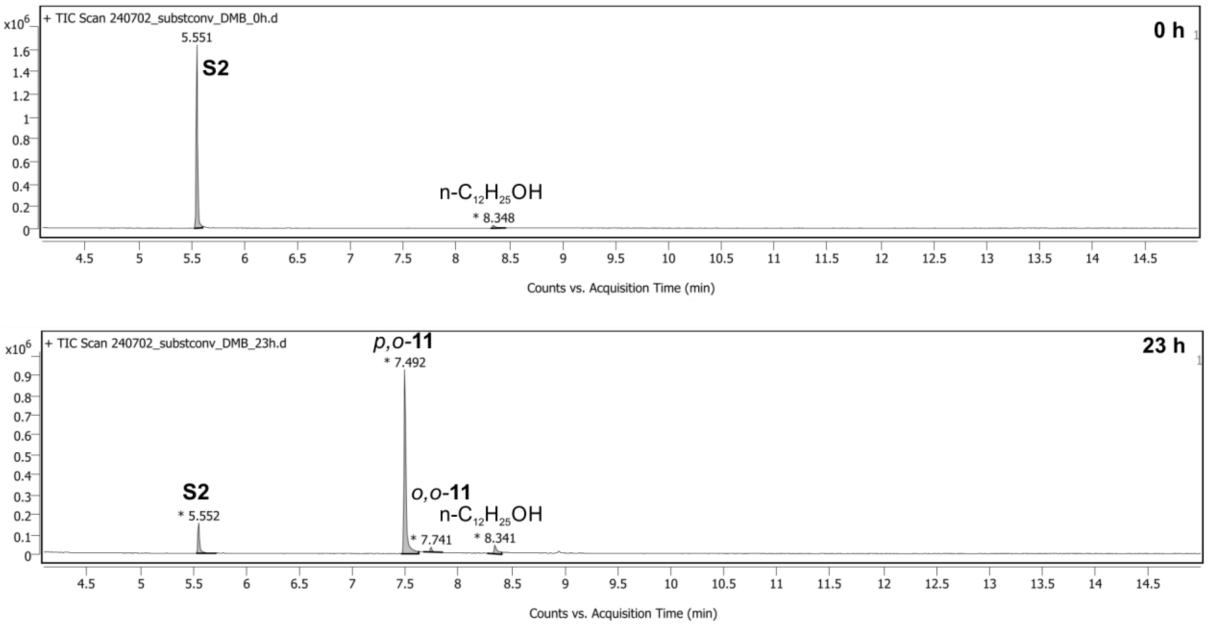

SFigure 31 | Chlorination of 1,3-dimethoxybenzene (S2) using *Am*VHPO-R425S

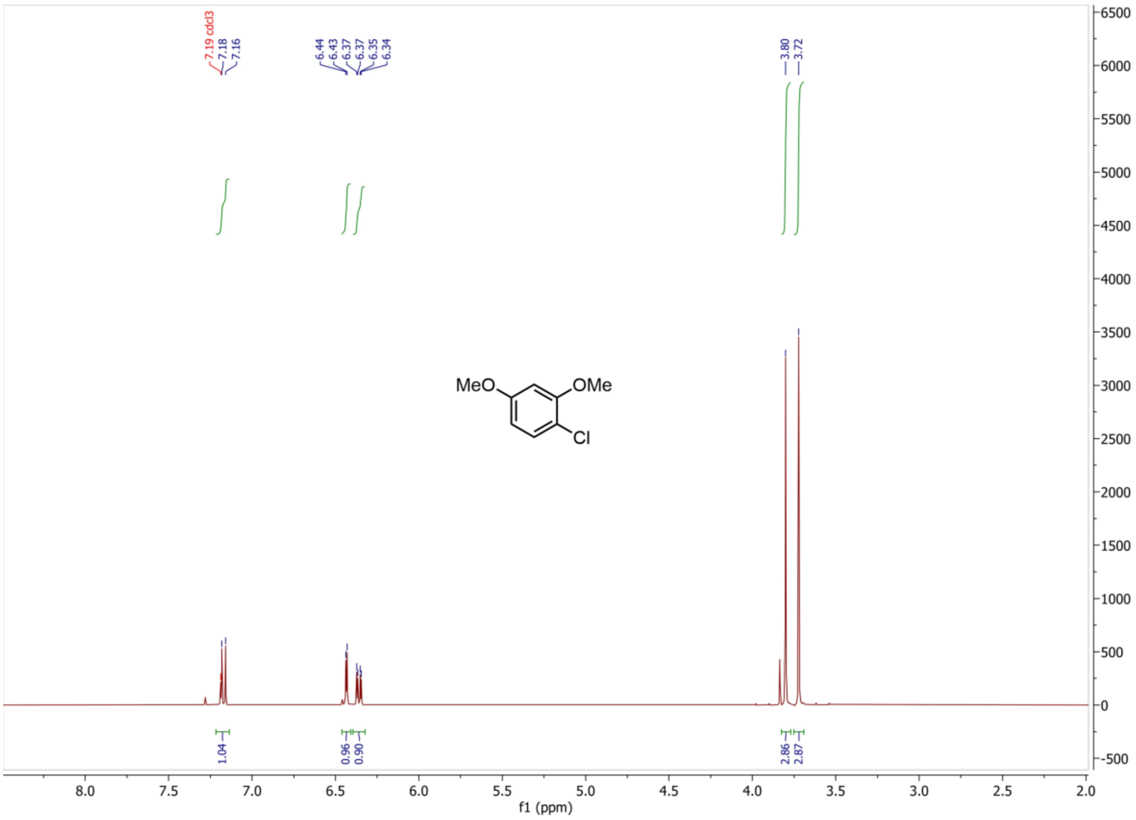

SFigure 32 | ^{1}H-NMR spectrum of product *p,o*-11

1-Chloro-2,4-dimethoxybenzene (*p,o*-11):[25] **^{1}H-NMR** (400 MHz, $CDCl_3$) δ = 7.18 (d, *J* = 8.7 Hz, 1H), 6.43 (d, *J* = 2.2 Hz, 1H), 6.36 (dd, *J* = 8.7, 2.7 Hz, 1H), 3.80 (s, 3H), 3.72 (s, 3H) ppm; **MS** (*p,o*-**11**, EI, 70eV): m/z (%) = 174 $[M]^+$ (8), 172 $[M]^+$ (26), 138 $[M]^+$ (100), 109 $[M]^+$ (36), 95 $[M]^+$ (23).

The physical and spectroscopic data agree with those reported in the literature.[25]

11.5 Chlorination of *N*,*N*-Dimethylaniline (S3)

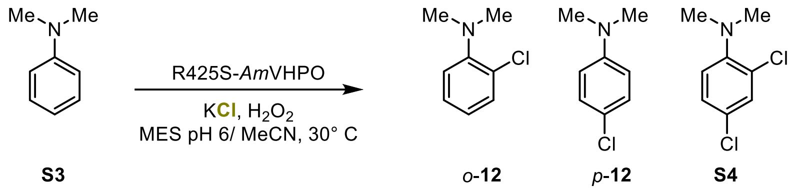

time	Conversion of **S3**	Ratio of *o*-**12**:*p*-**12**	Ratio of *o*-**12**:*p*-**12** (chemical conversion)	**Isolated yield** *o*-**12**
24 h	97%	87:13	62:38	80%

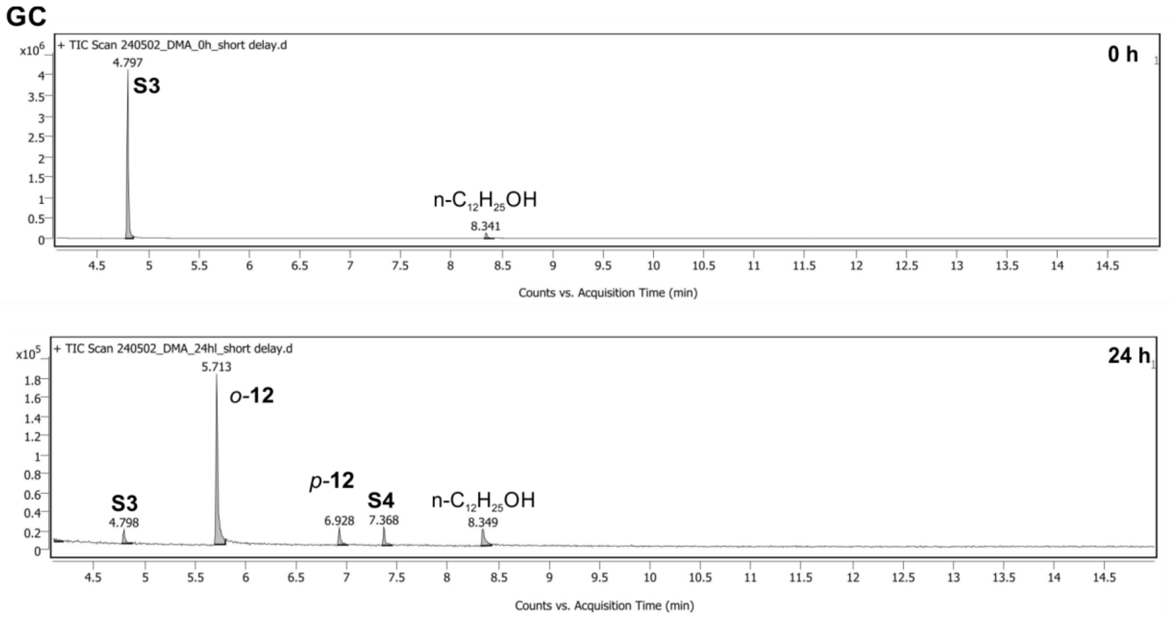

SFigure 33 | Chlorination of *N*,*N*-dimethylaniline (S3) using *Am*VHPO-R425S

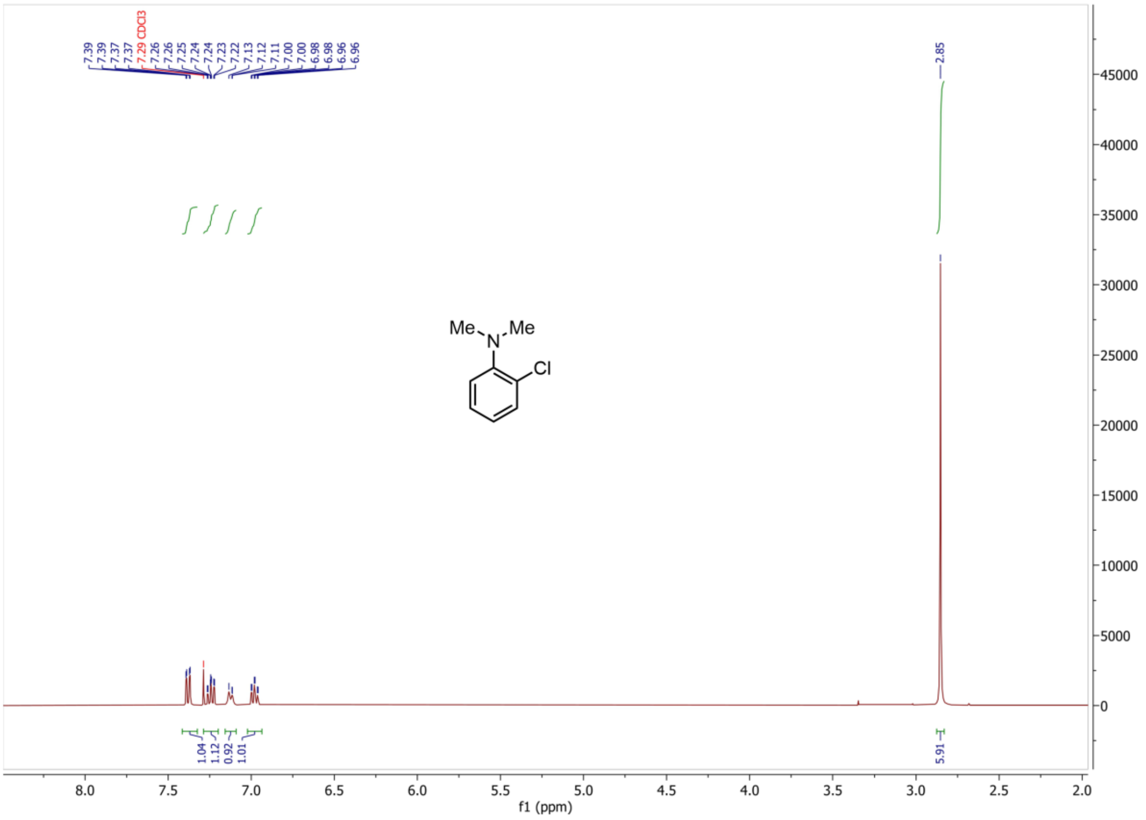

SFigure 34 | ^{1}H-NMR spectrum of product *o*-12

2-Chloro-*N,N*-dimethylaniline (*o*-12):[25] **^{1}H-NMR** (400 MHz, $CDCl_3$) δ = 7.38 (dd, *J* = 7.9, 1.6 Hz, 1H), 7.24 (ddd, *J* = 8.9, 7.3, 1.6 Hz, 1H), 7.12 (d, *J* = 8.5 Hz, 1H), 6.98 (td, *J* = 7.6, 1.6 Hz, 1H), 2.85 (s, 6H) ppm; **MS** (*o*-**12**, EI, 70eV): m/z (%) = 156 $[M]^+$ (11), 154 $[M]^+$ (36), 121 $[M]^+$ (31), 120 $[M]^+$ (30), 92 $[M]^+$ (34), 91 (58), 78 (100).

The physical and spectroscopic data agree with those reported in the literature.[25]

11.6 Chlorination of 3,5-dimethyl-*1H*-pyrrole-2-carboxylic acid ethyl ester (S5)

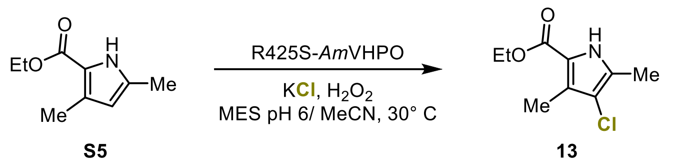

time	Conversion of **S5**	isolated yield of **13**
24 h	93%	81%

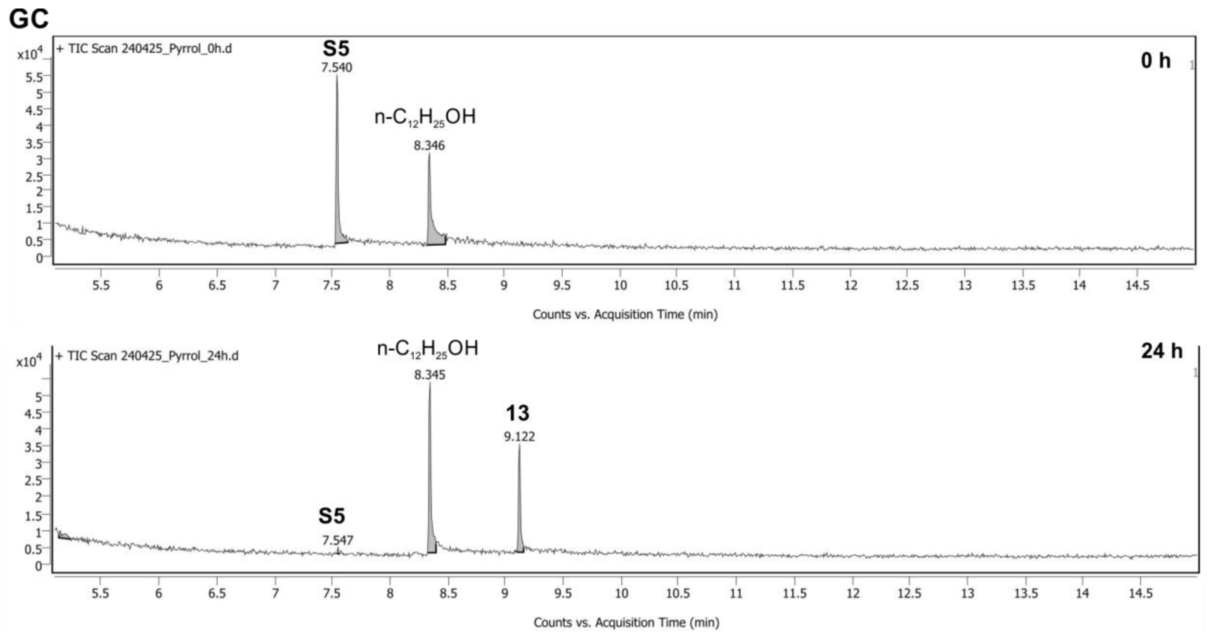

SFigure 35 | Chlorination of 3,5-dimethyl-1H-pyrrole-2-carboxylic acid ethyl ester (S5) using *Am*VHPO-R425S

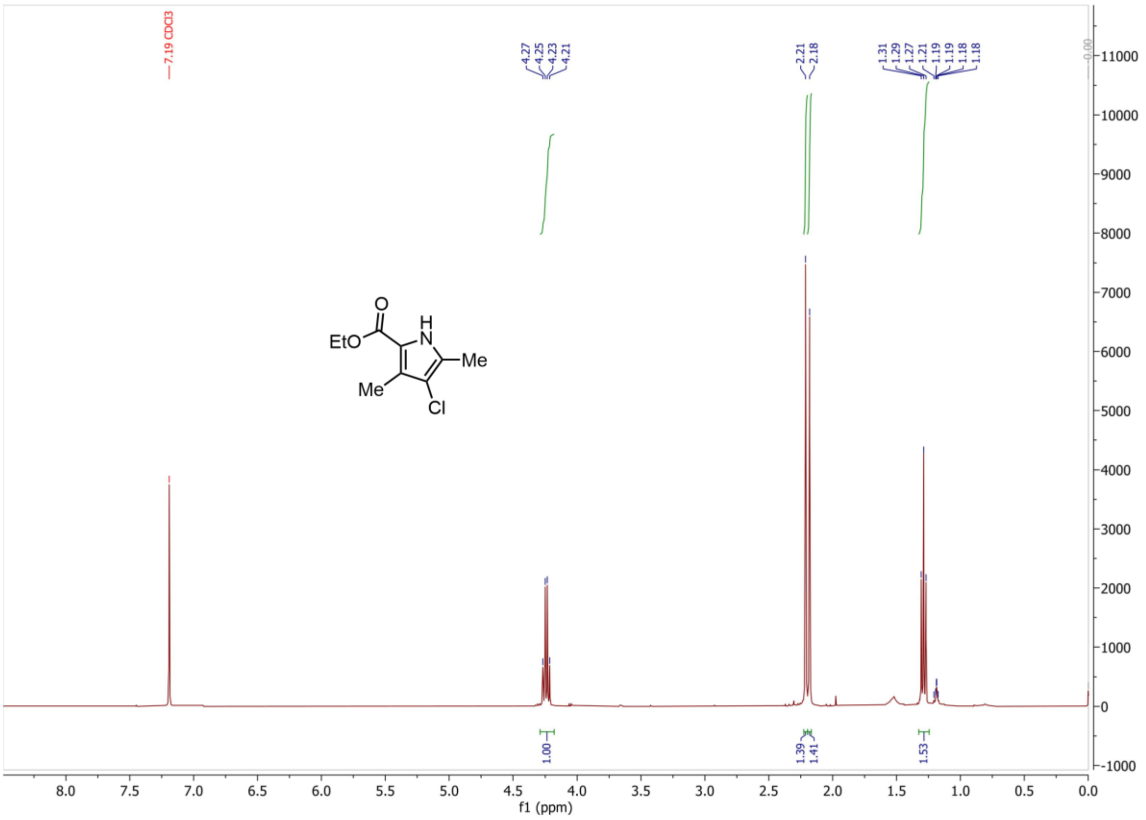

SFigure 36 | ^{1}H-NMR spectrum of product 13

Ethyl 4-chloro-3,5-dimethyl-1*H*-pyrrole-2-carboxylate (13):[25] **^{1}H-NMR** (400 MHz, $CDCl_3$) 4.24 (q, *J* = 7.1 Hz, 2H), 2.21 (s, 3H), 2.18 (s, 3H), 1.29 (t, *J* = 7.1 Hz, 3H) ppm; **MS** (**13**, EI, 70eV): m/z (%) = 203 $[M]^+$ (14), 201 $[M]^+$ (66), 156 $[M]^+$ (53), 155 $[M]^+$ (100), 92 $[M]^+$ (38), 91 (24), 78 (43), 65 (24).

The physical and spectroscopic data agree with those reported in the literature.[25]

11.7 Chlorination of 2-methylindole (S6)

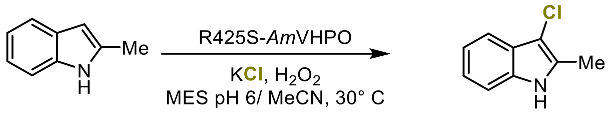

S6 **14**

time	Conversion of **S6**	Isolated yield of **14**
40 h	63%	49%

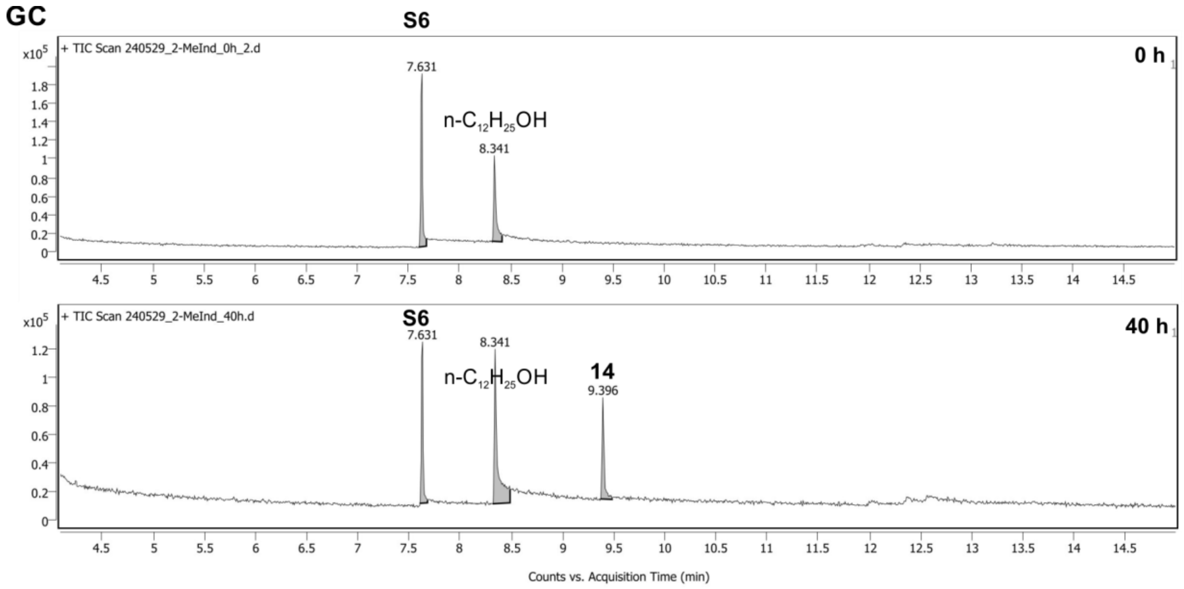

SFigure 37 | Chlorination of 2-methylindole (S6) using *Am*VHPO-R425S

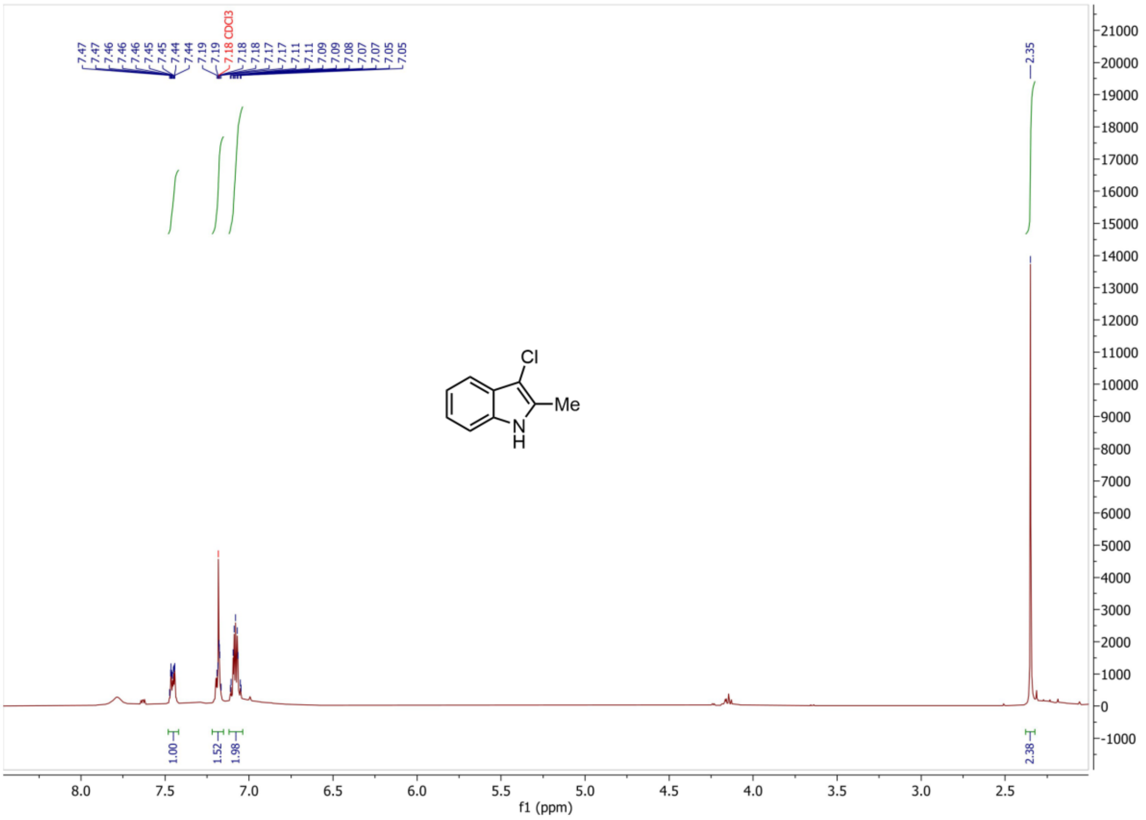

SFigure 38 | ¹H-NMR spectrum of product 14

3-Chloro-1-methyl-1*H*-indole (14):[25] **¹H-NMR** (400 MHz, $CDCl_3$) δ = 7.47 – 7.44 (m, 1H), 7.19 – 7.17 (m, 1H), 7.11 – 7.05 (m, 2H), 2.35 (s, 3H) ppm; **MS** (**14**, EI, 70eV): m/z (%) = 167 $[M]^+$ (29), 165 $[M]^+$ (100), 130 $[M]^+$ (69), 101 $[M]^+$ (13), 91 (38), 78 (77).

The physical and spectroscopic data agree with those reported in the literature.[25]

11.8 Chlorination of 2,6-dimethoxypyridine (S7)

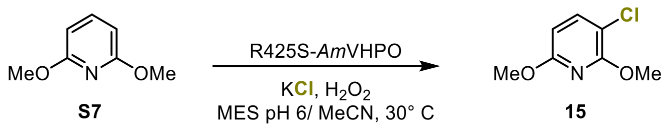

time	Conversion of **S7**	isolated yield of **15**
48 h	37%	21%

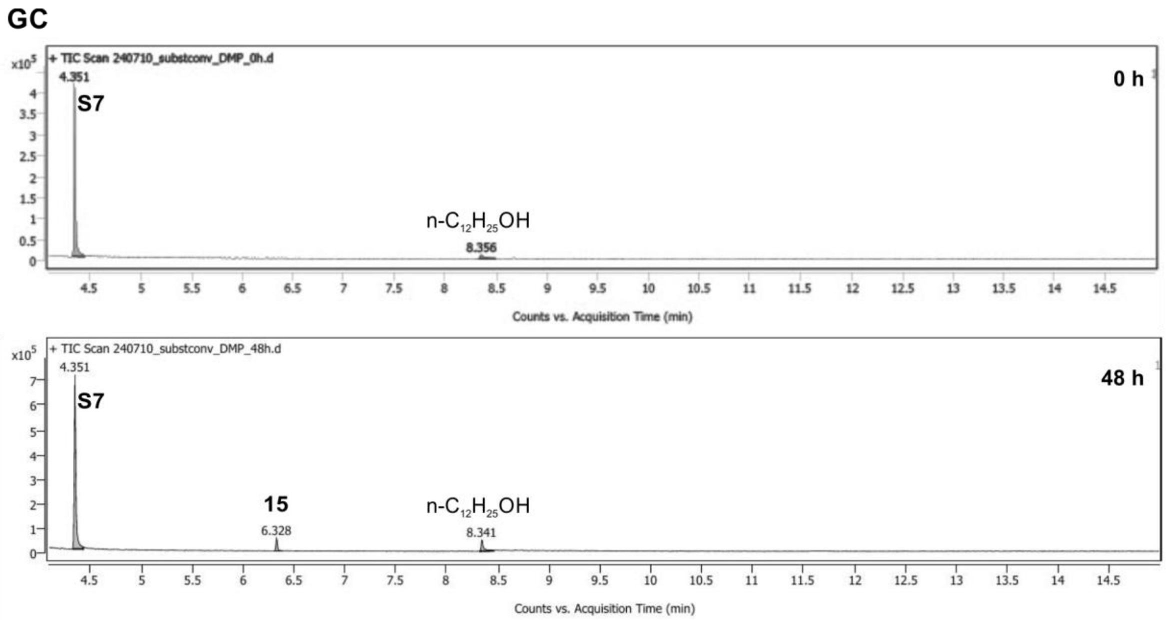

SFigure 39 | Chlorination of 2,6-dimethoxypyridine (S7) using *Am*VHPO-R425S

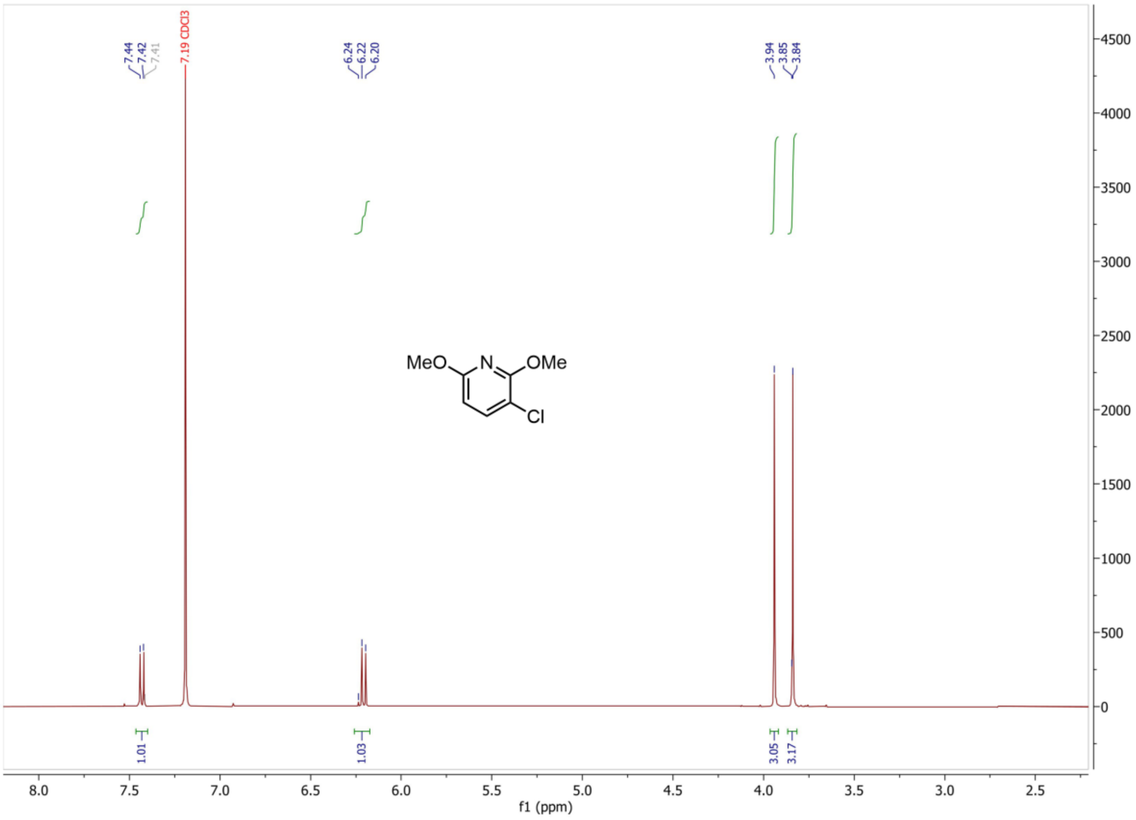

SFigure 40 | ^{1}H-NMR spectrum of product 15

3-Chloro-2,6-dimethoxypyridine (15):[25] **^{1}H-NMR** (400 MHz, $CDCl_3$) δ = 7.43 (d, *J* = 8.3 Hz, 1H), 6.22 (d, *J* = 8.4 Hz, 1H), 3.94 (s, 3H), 3.84 (s, 3H) ppm; **MS** (**15**, EI, 70eV): m/z (%) = 175 $[M]^+$ (33), 173 $[M]^+$ (100), 144 $[M]^+$ (53), 138 $[M]^+$ (61), 110 $[M]^+$ (23), 108 (83), 91 (52), 79 (94).

The physical and spectroscopic data agree with those reported in the literature.[25]

11.9 Chlorination of carbazole (S8)

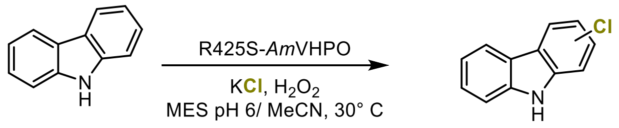

S8 **S9**

time	Conversion of **S8**
24 h	< 1%

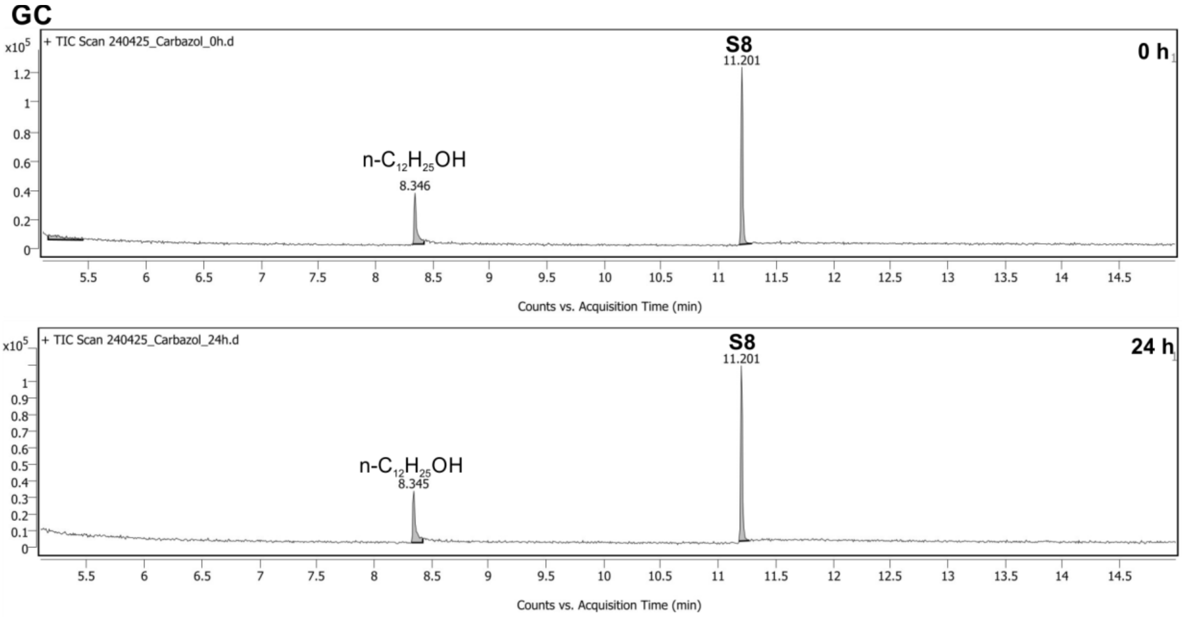

SFigure 41 | Chlorination of carbazole (S8) using *Am*VHPO-R425S

11.10 Chlorination of 3,5,4'-Trimethoxystilbene (S10)

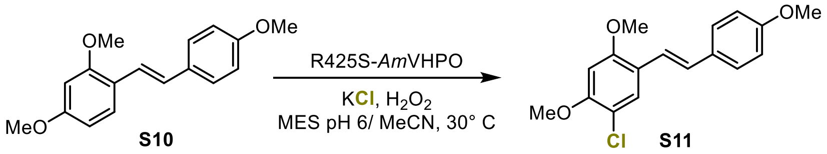

time	Conversion of **S10**
24 h	< 1%

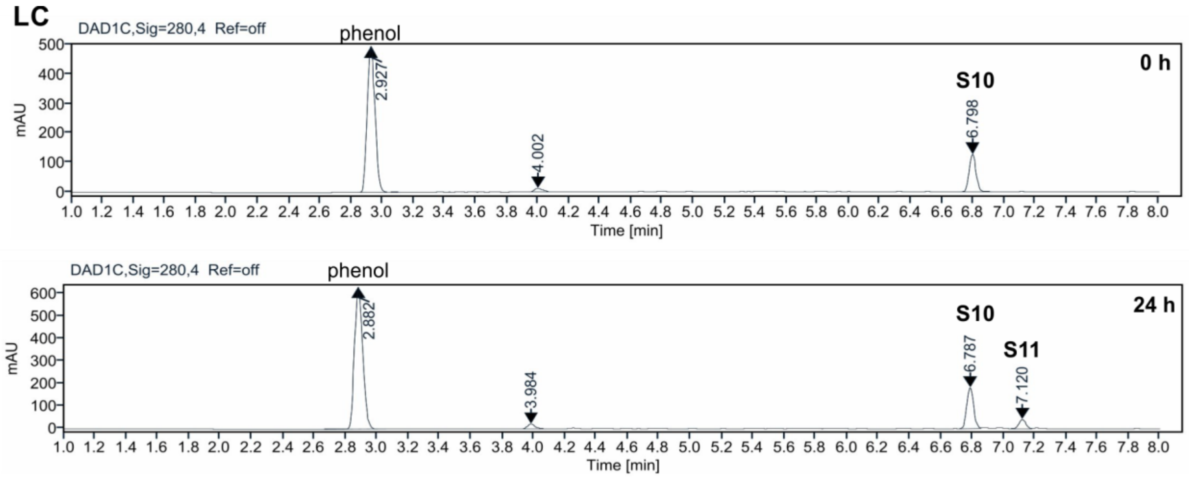

SFigure 42 | Chlorination of 3,5,4'-trimethoxystilbene (S10) using *Am*VHPO-R425S

12. CHEMICAL CHLORINATION

12.1 General Method

A round bottom flask equipped with a stirring bar was charged with the arene/heterocycle (0.22 mmol, 1.0 eq.), *N*-chlorosuccinimide (NCS, Alfa Aesar, A10310, 1.1 eq.), and DABCO (Sigma-Aldrich, D27802, 5-10 mol%). Dichloromethane (Acros, 8812283) (5 ml) was added. The reaction vessel was sealed and stirred at ambient temperature overnight. The crude product was purified by column chromatography using silica gel with ethyl acetate in hexane as the eluent. LC-MS data were obtained using OpenLab CDS Acquisition version 2.6 and OpenLab CDS Analysis version 2.6. GC-MS data were obtained using MassHunter Workstation GC/MS Data Acquisition version 10.2.489 and MassHunter Workstation Qualitative Analysis version 10.0. ^{1}H-NMR spectra are shown for the purified main products. NMR data were analyzed using MestReNova version 14.1.0-24037.

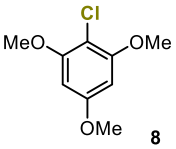

8

2-Chloro-1,3,5-trimethoxybenzene (8): white solid, 54% yield. ^{1}H NMR (400 MHz, $CDCl_3$) δ 6.21 (s, 2H), 3.90 (s, 6H), 3.83 (s, 3H) ppm.

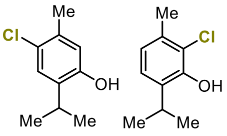

p-**10** *o*-**10**

4-chloro-5-methyl-2-(propan-2-yl)phenol (*p*-10) and 2-chloro-3-methyl-6-(propan-2-yl)phenol (*o*-10): 55:45 mixture of regioisomers, white solid, 31.3 mg, 77% yield. ^{1}H NMR (400 MHz, CDCl3) δ 7.73 (m, 0.5 H), 7.56 (m, 0.5 H), 7.03 (d, *J* = 9.8 Hz, 1 H), 6.79 (d, *J* = 6.1 Hz, 1 H), 4.25 (sep, *J* = 7.2 Hz, 1 H), 3.30 (sep, *J* = 8.8 Hz, 1 H), 2.44 (s, 3H), 2.36 (s, 3H), 1.26 (d, J = 4.2Hz, 6 H), 1.24 (d, J = 4.5 Hz, 6 H) ppm.

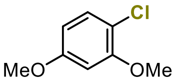

p,o-**11**

1-Chloro-2,4-dimethoxybenzene (*p*-10): colorless oil, 28.4 mg, 75% yield. 1H NMR (400 MHz, CDCl3) δ 7.24 (d, *J* = 10.9 Hz, 1H), 6.50 (d, *J* = 3.4 Hz, 1H), 6.43 (dd, *J* = 10.9, 3.4 Hz, 1H), 3.87 (s, 3H), 3.79 (s, 3H) ppm.

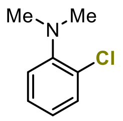

o-**12**

2-Chloro-*N*,*N*-dimethylaniline (*o*-12): colorless oil, 11.0 mg, 32% yield. ^{1}H NMR (400 MHz, CDCl3) δ 7.35 (dd, J = 9.9, 2.0 Hz, 1H), 7.22 (td, J = 9.9, 2.0 Hz, 1H), 7.13 (br), 6.96 (t, 9.8 Hz), 2.84 (s, 6H) ppm.

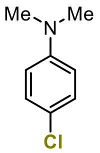

o-**12**

4-Chloro-*N*,*N*-dimethylaniline (*p*-12): colorless oil, 20% yield. ^{1}H NMR (400 MHz, $CDCl_3$) δ 7.20 (dt, J = 9.0, 3.5 Hz, 2H), 6.68 (d, J = 7.7Hz, 2H), 2.95 (s, 6H) ppm.

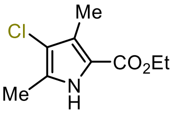

13

Ethyl-4-chloro-3,5-dimethyl-1*H*-pyrrole-2-carboxylate (13): light red solid, 28% yield. ^{1}H NMR (400 MHz, $CDCl_3$) δ 8.76 (s br, 1H), 4.31 (q, J = 7.1 Hz, 2H), 2.28 (s, 3H), 2.25 (s, 3H), 1.36 (t, J= 7.1 Hz, 3H) ppm.

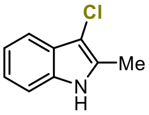

14

3-Chloro-2-methyl-1*H*-indole (14): red solid, 25% yield. ^{1}H NMR (400 MHz, $CDCl_3$) δ 7.86 (s br, 1H), 7.54 – 7.51 (m, 1H), 7.27 – 7.24 (m, 1H), 7.18 – 7.13 (m, 2H), 2.43 (s, 3H) ppm.

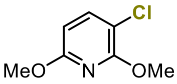

15

3-Chloro-2,6-dimethoxypyridine (15): colorless oil, 24.3 mg, 64% yield. 1H NMR (400 MHz, CDCl3) δ 7.52 (d, J = 10.3 Hz, 1H), 6.30 (d, J = 10.4 Hz, 1H), 4.03 (s, 3H), 3.93 (s, 3H) ppm.

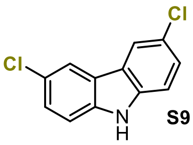

S9

3,6-Dibromo-9*H*-carbazole (S14): beige solid, 16% yield. ^{1}H NMR (400 MHz, $CDCl_3$) δ 8.09 (br s, 1H), 7.99 (d, J = 2.4 Hz, 2H), 7.52 (dd, J = 10.8, 5.2 Hz, 2H), 7.31 (d, J = 10.6 Hz, 2H) ppm.

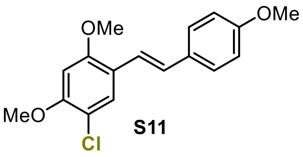

S11

1-Chloro-2,4-dimethoxy-5-[(*E*)-2-(4-methoxyphenyl)-ethenyl]-benzene (S13): white solid, 31% yield. ^{1}H NMR (400 MHz, $CDCl_3$) δ 7.50 (d, J = 11 Hz, 2H), 7.41 (d, J = 20.3 Hz, 1H), 7.00 (d, J = 20.3 Hz, 1H), 6.91 (d, J = 11 Hz, 2H), 6.80 (d, J = 3.5 Hz, 1H), 6.44 (d, J = 3.3 Hz, 1H), 3.89 (s, 3H), 3.86 (s, 3H), 3.84 (s, 3H) ppm.

The physical and spectroscopic data agree with those reported in the literature.[25]

13. SEQUENCE ALIGNMENT OF VHPOS

```
                      1                  20                   40
Am VHPO ..............MNTRRQQAQNIRNNAAELAAN.RPHPQHINNKEEYEYRRPKKDGNE
Cp VHPO .......MGIPADNLQSRAKASFDTRVAAAELALN.RGVVPSFANGEELLYRNPDPDNTD
Co VHPO .......MGIPADNLQSRAKASFDTRVAAAELALA.RGAVPSFANGEELLYRNSE..TGD
An VHPO QTCSTSDDADDPTPPNERDDEAFASRVAAAKRELEGTGTVC.............QINNGE
Ci VHPO ............................................................

                      60                  80                  100
Am VHPO PSHIANFTKGLPHDEHTGLLLNSADYDQFVLGIQSGDTTDFARTPLGPAELPKVHGCLSK
Cp VHPO PSFIASFTKGLPHDDN.GAIIDPDDFLAFVRAINSGDEKEIADLTLGPARDPET......
Co VHPO PSFIGSFTKGLPHDDN.GAIIDPDDFLAFVRAINSGDEKEIAALTLGPARDPET......
An VHPO TDLAAKFHKSLPHDDLGQ..VDADAFAALEDCILNGDLSICEDVPVGNS...........
Ci VHPO .....................................MGSVTPIPLPKIDEPEE......

                      120                 140                 160
Am VHPO QKIDCDDDHRSGFWKSQIAQGAAGGDGAKLRAWESAGAGLVFDLEGPDAQAVTMPPAPRL
Cp VHPO .........GLPIWRSDLAN....SLELEVRGWENSSAGLTFDLEGPDAQSIAMPPAPVL
Co VHPO .........GLPIWRSDLAN....SLDLEVRGWENSSAGLTFDLEGPDAQSVAMPPAPVL
An VHPO .........................EGDPVGRLVNPTAAFAIDISGPAFSATTIPPVPTL
Ci VHPO .....YNTNYILFWNHVGLE............................LNRVTHTVGGPL

                              180                 200
Am VHPO ESPEL........TSEIAEVYSQALLRDIHFSQLRDPGLGDQVNACDSCPTQLSIYEAID
Cp VHPO TSPEL........VAEIAELYLMALGREIEFSEFDSPKNAEYIQ................
Co VHPO TSPEL........IAEMAELYLMALGRDIEFSEFDSPKNAAFIR................
An VHPO PSPEL........AAQLAEVYWMALARDVPFMQYGTDDITVTA.................
Ci VHPO TGPPLSARALGMLHLAIHDAYF.SICPPTDFTTFLSPDTENA...AYRLPSPNGANDARQ

          220                          240                 260
Am VHPO ILNTVQIEGQ..NWFSANCCDL..T.....DDEQARQRPLVTRQNIFRGIAPGDDVGPYL
Cp VHPO .FAIDQLNGL..EWFNTPAKLG.DP.....PAEIRRRRGEVTVGNLFRGILPGSEVGPYL
Co VHPO .SAIERLNGL..EWFNTPAKLG.DP.....PAEIRRRRGEVTVGNLFRGILPGSEVGPYL
An VHPO ...AANLAGM..EGFPNLDA...........VSIGSDGTVDPLSQLFRATFVGVETGPFI
Ci VHPO AVAGAALKMLSSLYMKPVEQPNPNPGANISDNAYAQLGLVLDRSVLE...APGGVDRE..

                   280                             300
Am VHPO SQFLLIGNNALGGGVFGQ...........EAGHIGYGAIRIDQRVRKATPCKDFM.....
Cp VHPO SQYIIVGSKQIGSATVGNKTLVSPNAADEFDGEIAYGSITISQRVRIATPGRDFM.....
Co VHPO SQFIIVGSKQIGSATVGNKTLVSPNAADEFDGEIAYGSITISQRVRIATPGRDFM.....
An VHPO SQLLVNS..........................FTIDSITVEPKQETFAPDVNYM.....
Ci VHPO SASFMFG..........E......DVA...DVFFA...LLNDPR......GASQEGYHPT

                            320                    340
Am VHPO ......TNFETWL.......DVQNGADLRGLETYVDAD...PGK......CREFPAYRVI
Cp VHPO ......TDLKVFL.......DVQDAADFRGFESYE...................PGARLI
Co VHPO ......TDLKVFL.......DVQDAADFRGFESYE...................PGARLI
An VHPO ......VDFDEWL.......NIQNGGPPAGPELLD...................DELRFV
Ci VHPO PGRYKFDDEPTHPVVLIPVDPNNPNGPKMPFRQYHAPFYGKTTKRFATQSEHFLADPPGL

                 360                                          380
Am VHPO TTPRDLATYVHYDALYEAYL.NACL........................ILLGMGAPFDP
Cp VHPO RTIRDLATWVHFDALYEAYL.NACL........................ILLANGVPFDP
Co VHPO RTIRDLATWVHFDSLYEAYL.NACL........................ILLANGVPFDP
An VHPO RNARDLARVTFTDNINTEAY.RGAL........................ILLGLDAFNRA
Ci VHPO RSN..........ADETAEYDDAVRVAIAMGGAQALNSTKRSPWQTAQGLYWAYDGSNLI

                                   400                 420
Am VHPO GIPF.............QKPDVEDKQQGFAHFGGPQILTLVCEAATRGLKAVRFQKFNVH
Cp VHPO NLPF.............QQEDKLDNQDVFVNFGSAHVLSLVTEVATRALKAVRYQKFNIH
Co VHPO NLPF.............QQEDKLDNQDVFVNFGSAHVLSLVTEVATRALKAVRYQKFNIH
An VHPO GVNG..............PFIDIDRQAGFVNFGISHYFRLIGA.AELAQRSSWYQKWQVH
Ci VHPO GTPPRFYNQIVRRIAVTYKKEEDLANSEVNNADFARLFALVDVACTDAGIFSWKEKWE..

               440                 460                 480
Am VHPO RRLRPEALGGLVDRYKHGKGAGDELKPVAALVEALENVGLLSKVVAHNQLQNQNLDRSGD
Cp VHPO RRLRPEATGGLISVNKIAAQKGESIFPEVDLA..VEEL...GDILEKAEISNRKQNIADG
Co VHPO RRLRPEATGGLISVNKNAFLKSESVFPEVDVL..VEEL...SSILDDSASSNEKQNIADG
An VHPO RFARPEALGGTLHLTIKGELNADFDLS......LLENAELLKRVAAIN.........AAQ
Ci VHPO .....................FEFWRPLSGVR.........................DDG
```

```
                  500                       520                     540
                    |                         |                       |
Am VHPO  PSSAGDNYFLPMAFPE.......GSPMHPSYGAGHATVAGACVTMLKAFFDHG...WQLN
Cp VHPO  DPDPDPSFLLPMAFAE.......GSPFHPSYGSGHAVVAGACVTILKAFFDSG...IEID
Co VHPO  DVSPGKSFLLPMAFAE.......GSPFHPSYGSGHAVVAGACVTILKAFFDAN...FQID
An VHPO  NPNNEVTYLLPQAIQE.......GSPTHPSYPSGHATQNGAFATVLKALIGLD...RGGD
Ci VHPO  RPDHGDPFWLTLGAPATNTNDIPFKPPFPAYPSGHATFGGAVFQMVRRYYNGRVGTWKDD

                                        560                  580
                                          |                    |
Am VHPO  LGMANGKY..............I.SYEPNQDGSSLQQVLLD.CPLTVEGELNKIAANISI
Cp VHPO  QV.......................FEVDKDEDKLVKSSFK.GTLTVAGELNKLADNIAI
Co VHPO  QV.......................FEVDTDEDKLVKSSFP.GPLTVAGELNKLADNVAI
An VHPO  CY.....P..............D.PVYPDDDGLKLIDF.RG.SCLTFEGEINKLAVNVAF
Ci VHPO  EPDNIAIDMMISEELNGVNRDLRQPYDPTAPIEDQPGIVRTRIVRHFDSAW.ELMFENAI

                       600                       620
                         |                         |
Am VHPO  GRDWAGVHYFTDYIESLRLGEKIA..I.GILEE...QKLTYGENFTMTVPLYDGGSIQI.
Cp VHPO  GRNMAGVHYFSDQFESLLLGEQVA..I.GILEE...QSLTYGENFFFNLPKFDGTTIQI.
Co VHPO  GRNMAGVHYFSDQFESLLLGEQIA..I.GILEE...QSLTYGENFFFNLPKFDGTTIQI.
An VHPO  GRQMLGIHYRFDGIQGLLLGETIT..V.RTLHQ...ELMTFAEESTFEFRLFTGEVIKLF
Ci VHPO  SRIFLGVHWRFDAAAARDILIPTTTKDVYAVDNNGATVFQNVEDIRYTTRGTRED.....

Am VHPO  ............................................................
Cp VHPO  ............................................................
Co VHPO  ............................................................
An VHPO  QDGTFTIDGFKCP.....GLVYTGVENCV...............................
Ci VHPO  EEGLFPIGGVPLGIEIADEIFNNGLKPTPPEIQPMPQETPVQKPVGQQPVKGMWEEEQAP

Am VHPO  ......
Cp VHPO  ......
Co VHPO  ......
An VHPO  ......
Ci VHPO  VVKEAP
```

SFigure 43 | Sequence alignment of various VHPOs to determine important amino acids for halogen specificity.[1,8-12] Fully conserved residues are highlighted in red bars.

14. REFERENCES

1 Renirie, R., Pierlot, C., Aubry, J. M., Hartog, A. F., Schoemaker, H. E., Alsters, P. L. & Wever, R. Vanadium chloroperoxidase as a catalyst for hydrogen peroxide disproportionation to singlet oxygen in mildly acidic aqueous environment. *Adv Synth Catal* **345**, 849-858, doi:DOI 10.1002/adsc.200303008 (2003).

2 Frank, A., Seel, C. J., Groll, M. & Gulder, T. Characterization of a cyanobacterial haloperoxidase and evaluation of its biocatalytic halogenation potential. *ChemBioChem* **17**, 2028-2032, doi:10.1002/cbic.201600417 (2016).

3 Ohshiro, T., Littlechild, J., Garcia-Rodriguez, E., Isupov, M. N., Iida, Y., Kobayashi, T. & Izumi, Y. Modification of halogen specificity of a vanadium-dependent bromoperoxidase. *Protein Sci.* **13**, 1566-1571, doi:https://doi.org/10.1110/ps.03496004 (2004).

4 Soedjak, H. S. & Butler, A. Chlorination catalyzed by vanadium bromoperoxidase. *Inorg. Chem.* **29**, 5015-5017, doi:10.1021/ic00350a003 (1990).

5 van Schijndel, J. W. P. M., Vollenbroek, E. G. M. & Wever, R. The chloroperoxidase from the fungus Curvularia inaequalis; a novel vanadium enzyme. *Biochim. Biophys. Act.* **1161**, 249-256, doi:https://doi.org/10.1016/0167-4838(93)90221-C (1993).

6 Jumper, J., Evans, R., Pritzel, A., Green, T., Figurnov, M., Ronneberger, O., Tunyasuvunakool, K., Bates, R., Žídek, A., Potapenko, A., Bridgland, A., Meyer, C., Kohl, S. A. A., Ballard, A. J., Cowie, A., Romera-Paredes, B., Nikolov, S., Jain, R., Adler, J., Back, T., Petersen, S., Reiman, D., Clancy, E., Zielinski, M., Steinegger, M., Pacholska, M., Berghammer, T., Bodenstein, S., Silver, D., Vinyals, O., Senior, A. W., Kavukcuoglu, K., Kohli, P. & Hassabis, D. Highly accurate protein structure prediction with AlphaFold. *Nature* **596**, 583-589, doi:10.1038/s41586-021-03819-2 (2021).

7 Leaver-Fay, A., Tyka, M., Lewis, S. M., Lange, O. F., Thompson, J., Jacak, R., Kaufman, K., Renfrew, P. D., Smith, C. A., Sheffler, W., Davis, I. W., Cooper, S., Treuille, A., Mandell, D. J., Richter, F., Ban, Y.-E. A., Fleishman, S. J., Corn, J. E., Kim, D. E., Lyskov, S., Berrondo, M., Mentzer, S., Popović, Z., Havranek, J. J., Karanicolas, J., Das, R., Meiler, J., Kortemme, T., Gray, J. J., Kuhlman, B., Baker, D. & Bradley, P. ROSETTA3: an object-oriented software suite for the simulation and design of macromolecules. *Methods Enzymol.* **487**, 545-574, doi:10.1016/B978-0-12-381270-4.00019-6. (2011).

8 DiMaio, F., Leaver-Fay, A., Bradley, P., Baker, D. & André, I. Modeling Symmetric Macromolecular Structures in Rosetta3. *PLOS ONE* **6**, e20450, doi:10.1371/journal.pone.0020450 (2011).

9 Dauparas, J., Anishchenko, I., Bennett, N., Bai, H., Ragotte, R. J., Milles, L. F., Wicky, B. I. M., Courbet, A., de Haas, R. J., Bethel, N., Leung, P. J. Y., Huddy, T. F., Pellock, S., Tischer, D., Chan, F., Koepnick, B., Nguyen, H., Kang, A., Sankaran, B., Bera, A. K., King, N. P. & Baker, D. Robust deep learning–based protein sequence design using ProteinMPNN. *Science* **378**, 49-56, doi:10.1126/science.add2187 (2022).

10 Cock, P. J. A., Antao, T., Chang, J. T., Chapman, B. A., Cox, C. J., Dalke, A., Friedberg, I., Hamelryck, T., Kauff, F., Wilczynski, B. & de Hoon, M. J. L. Biopython: freely available Python tools for computational molecular biology and bioinformatics. *Bioinformatics* **25**, 1422-1423, doi:10.1093/bioinformatics/btp163 (2009).

11 Shrake A Fau - Rupley, J. A. & Rupley, J. A. Environment and exposure to solvent of protein atoms. Lysozyme and insulin. *J. Mol. Biol.* **15**, 351-371, doi:doi: 10.1016/0022-2836(73)90011-9. (1973).

12 Frenz, B., Lewis, S. M., King, I., DiMaio, F., Park, H. & Song, Y. Prediction of Protein Mutational Free Energy: Benchmark and Sampling Improvements Increase Classification Accuracy. *Front. Bioeng. Biotechnol.* **8**, doi:org/10.3389/fbioe.2020.558247 (2020).

13 Goddard, T. D., Huang, C. C., Meng, E. C., Pettersen, E. F., Couch, G. S., Morris, J. H. & Ferrin, T. E. UCSF ChimeraX: Meeting modern challenges in visualization and analysis. *Protein Sci.* **27**, 14-25, doi:https://doi.org/10.1002/pro.3235 (2018).

14 Gérard, E. F., Mokkawes, T., Johannissen, L. O., Warwicker, J., Spiess, R. R., Blanford, C. F., Hay, S., Heyes, D. J. & de Visser, S. P. How Is Substrate Halogenation Triggered by the Vanadium Haloperoxidase from Curvularia inaequalis? *ACS Catal.* **13**, 8247-8261, doi:10.1021/acscatal.3c00761 (2023).
15 Schwarten, M., Weiergraber, O. H., Petrovic, D., Strodel, B. & Willbold, D. Structural Studies of Autophagy-Related Proteins. *Methods Mol Biol* **1880**, 17-56, doi:10.1007/978-1-4939-8873-0_2 (2019).
16 Abraham, M. J., Murtola, T., Schulz, R., Pall, S., Smith, J. C., Hess, B. & Lindahl, E. GROMACS: High performance molecular simulations through multi-level parallelism from laptops to supercomputers. *SoftwareX* **1-2**, 19-25 (2015).
17 Lindorff-Larsen, K., Piana, S., Palmo, K., Maragakis, P., Klepeis, J. L., Dror, R. O. & Shaw, D. E. Improved side-chain torsion potentials for the Amber ff99SB protein force field. *Proteins* **78**, 1950-1958, doi:https://doi.org/10.1002/prot.22711 (2010).
18 MacKerell, A. D., Jr., Bashford, D., Bellott, M., Dunbrack, R. L., Jr., Evanseck, J. D., Field, M. J., Fischer, S., Gao, J., Guo, H., Ha, S., Joseph-McCarthy, D., Kuchnir, L., Kuczera, K., Lau, F. T. K., Mattos, C., Michnick, S., Ngo, T., Nguyen, D. T., Prodhom, B., Reiher, W. E., Roux, B., Schlenkrich, M., Smith, J. C., Stote, R., Straub, J., Watanabe, M., Wiórkiewicz-Kuczera, J., Yin, D. & Karplus, M. All-Atom Empirical Potential for Molecular Modeling and Dynamics Studies of Proteins. *J. Phys. Chem. B* **102**, 3586-3616, doi:10.1021/jp973084f (1998).
19 Beyer, J. N., Hosseinzadeh, P., Gottfried-Lee, I., Van Fossen, E. M., Zhu, P., Bednar, R. M., Karplus, P. A., Mehl, R. A. & Cooley, R. B. Overcoming Near-Cognate Suppression in a Release Factor 1-Deficient Host with an Improved Nitro-Tyrosine tRNA Synthetase. *J. Mol. Biol.* **432**, 4690-4704, doi:https://doi.org/10.1016/j.jmb.2020.06.014 (2020).
20 Bernetti, M. & Bussi, G. Pressure control using stochastic cell rescaling. *J. Chem. Phys.* **153**, 114107, doi:10.1063/5.0020514 (2020).
21 Pedregosa, F., Varoquaux, G., Gramfort, A., Michel, V., Thirion, B., Grisel, O., Blondel, M., Prettenhofer, P., Weiss, R., Dubourg, V., Vanderplas, J., Passos, A., Cournapeau, D., Brucher, M., Perrot, M. & Duchesnay, E. *J. Mach. Learn. Res.*, 2825-2830 (2011).
22 Kabsch, W. XDS. *Acta Crystallogr. D* **66**, 125-132, doi:doi:10.1107/S0907444909047337 (2010).
23 McCoy, A. J. G.-K., R. W.; Adams, P. D.; Winn, M. D.; Storoni, L. C.; Read, R. J. Phaser crystallographic software. *J. Appl. Crystallogr.* **40**, 658-674. (2007).
24 Emsley, P. & Cowtan, K. Coot: model-building tools for molecular graphics. *Acta Cryst. Sect. D* **60**, 2126-2132, doi:doi:10.1107/S0907444904019158 (2004).
25 Seel, C. J., Kralik, A., Hacker, M., Frank, A., Koenig, B. & Gulder, T. Atom-Economic Electron Donors for Photobiocatalytic Halogenations. *ChemCatChem* **10**, 3960-3963, doi:10.1002/cctc.201800886 (2018).
